# Magnesium and Hypertension in Old Age

**DOI:** 10.3390/nu13010139

**Published:** 2020-12-31

**Authors:** Ligia J. Dominguez, Nicola Veronese, Mario Barbagallo

**Affiliations:** Geriatric Unit, Department of Medicine, University of Palermo, 90100 Palermo, Italy; ligia.dominguez@unipa.it (L.J.D.); nicola.veronese@unipa.it (N.V.)

**Keywords:** magnesium, hypertension, aging, ions, insulin resistance, cardiovascular disease, diet, supplement

## Abstract

Hypertension is a complex condition in which various actors and mechanisms combine, resulting in cardiovascular and cerebrovascular complications that today represent the most frequent causes of mortality, morbidity, disability, and health expenses worldwide. In the last decades, there has been an exceptional amount of experimental, epidemiological, and clinical studies confirming a close relationship between magnesium deficit and high blood pressure. Multiple mechanisms may help to explain the bulk of evidence supporting a protective effect of magnesium against hypertension and its complications. Hypertension increases sharply with advancing age, hence older persons are those most affected by its negative consequences. They are also more frequently at risk of magnesium deficiency by multiple mechanisms, which may, at least in part, explain the higher frequency of hypertension and its long-term complications. The evidence for a favorable effect of magnesium on hypertension risk emphasizes the importance of broadly encouraging the intake of foods such as vegetables, nuts, whole cereals and legumes, optimal dietary sources of magnesium, avoiding processed food, which are very poor in magnesium and other fundamental nutrients, in order to prevent hypertension. In some cases, when diet is not enough to maintain an adequate magnesium status, magnesium supplementation may be of benefit and has been shown to be well tolerated.

## 1. Introduction

Magnesium is the most present divalent intracellular cation in the human body, and the second intracellular ion after potassium. This primary cation has been traditionally considered as cofactor of about 300 regulatory enzymes [1], but current databases list over 600 enzymes for which magnesium is cofactor [2]. Magnesium is involved in fundamental cellular reactions comprising ATP-dependent biochemical processes as part of the activated MgATP complex, DNA synthesis, RNA expression, muscular and neural cell signaling, glucose metabolism, and blood pressure control [3,4].

Although magnesium was first recommended as treatment for malignant hypertension as early as 1925 [5], subsequent studies failed to demonstrate reliable results. In 1983, a study by Resnick et al. [6], showed a close inverse relationship of serum ionized magnesium and plasma renin activity. The following year (1984) Resnick and Gupta, using novel ^31^P-NMR technique that allowed precise assessment of intracellular cytosolic magnesium concentrations, published a seminal paper showing that persons with essential hypertension had consistently lower levels of intracellular magnesium with an inverse relationship between these concentrations and blood pressure values, the lower intracellular magnesium, the higher blood pressure [7]. This close quantitative relationship confirmed the presence of a powerful link between magnesium deficiency and human essential hypertension. Afterwards, a number of experimental, clinical, and epidemiological studies exploring the relationship of this key cation with hypertension have been undertaken. Magnesium is involved in blood pressure regulation by diverse mechanisms including modulation of vascular tone and reactivity acting as a calcium antagonist [8,9], the renin–angiotensin–aldosterone system (RAAS) [6], endothelial function [10,11,12], vascular remodeling and stiffness [13], and catecholamine release [14]. Magnesium deficiency has been also related to low-grade inflammation, oxidative stress [12,15,16], insulin resistance, and metabolic syndrome [3].

High blood pressure is the strongest independent and modifiable risk factor for heart failure, ischemic heart disease, cerebrovascular events, chronic kidney disease, and cognitive decline worldwide [17]. Hypertension was associated with 4.9, 2.0, and 1.5 million deaths due to ischemic heart disease, hemorrhagic stroke, and ischemic stroke, respectively, in 2015 [18]. According to the World Health Organization, 1.13 billion adults have hypertension currently [19]. The prevalence of hypertension rises remarkably with advancing age and due to the continuous and global increase in aging populations, the prevalence of hypertension and its derived detrimental consequences are still increasing [20]. Hence, public health preventive actions are urgently needed, comprising nutrition, to combat the hypertension pandemic.

A number of investigations have assessed the association of dietary and supplemental magnesium with the development of high blood pressure and meta-analyses on cohort studies and RCTs have confirmed protective effects [21,22,23,24]. A recent summary of meta-analyses on the effects of electrolytes on hypertension revealed that the greatest beneficial effect on blood pressure lowering was ascribed to magnesium intake followed by potassium intake and by salt reduction [25]. Dietary magnesium intake is deficient in a large proportion of European and US populations where Western dietary patterns full of processed food are very frequent [26,27,28,29]. Indeed, magnesium is abundant in green leafy vegetables, nuts, legumes, and whole cereals, while it is practically absent in processed food and sugar sweetened beverages [30]. Chronic inadequate magnesium intake, particularly frequent in old age, has been associated with an increased risk of multiple clinical conditions including hypertension and stroke [3,8,31]. The Dietary Guidelines for Americans recommend a daily intake of 420 mg of magnesium for men and 320 mg for women [32], but estimates indicate that more than 60% of Americans are under the recommendation [28]. 

Most studies have shown inverse associations of dietary magnesium intake with hypertension [33,34,35] or risk of incident hypertension [36,37,38,39,40], while fewer studies have reported negative or inconclusive results [41,42,43]. A systematic review and meta-analysis of cohort studies reported that a 100 mg/day increment of magnesium dietary intake was significantly associated with 5% reduction in incident hypertension [21]. Three meta-analyses of 11, 34, and 28 RCTs found that supplementation with oral magnesium resulted in significant reductions in blood pressure vs. controls [22,23,44]. Earlier meta-analyses suggested benefit with less prominent effect, possibly due to heterogeneity and to the inclusion of persons with and without other chronic diseases in the analyses [24,45].

The present article aims to review the role of alterations of magnesium metabolism in the pathophysiology of high blood pressure, condition which is particularly frequent in old age. We discuss the possible mechanisms involved and the available evidence of the effects of dietary and supplemental magnesium on blood pressure lowering and risk of hypertension.

## 2. Magnesium Metabolism, Dietary Sources, and Requirements

Approximately 24 g (1 mole) of magnesium are present in the human body, of which almost 2/3 stored in the bone and 1/3 in the cellular compartment. Blood serum contains less than 1% of the total body magnesium with normal concentrations ranging between 0.75 and 0.95 mmol/L (1.7–2.5 mg/dL or 1.5–1.9 meq/L). Magnesium concentrations in the serum are extremely constant and are tightly controlled and kept within this narrow range by the kidney and small intestine increasing their fractional magnesium absorption during magnesium deprivation. If the lack of magnesium persists, bone stores help maintaining serum magnesium concentration through exchange with extracellular fluid [3].

Serum magnesium exists in three forms: 25% is bound to albumin and 8% bound to globulins (protein-bound fraction); 12% corresponds to the chelated fraction; while 55% represents the metabolically active ionized fraction. Hypomagnesemia is generally identified as a serum magnesium concentration below 0.75 mmol/L [3]. Magnesium is an intracellular regulator of the cell cycle physiology and apoptosis; its intracellular concentrations are as well highly regulated. Most intracellular magnesium exists in bound form. Circulating magnesium concentrations do not always correspond to intracellular or total magnesium.

Magnesium equilibrium depends on magnesium intake, its absorption through intestine (mainly small intestine), its renal excretion, and its requirements in all tissues [46]. Magnesium’s requirement per day in healthy adults is estimated at 300–400 mg (5 to 6 mg/kg/day) but in several physiological conditions this requirement may be increased (i.e., exercise, aging, pregnancy, etc.), as well as in some pathological conditions (diabetes, infections, etc.). Because magnesium stored in bone tissue cannot be quickly exchanged with magnesium in the extracellular fluids, the rapid magnesium needs are provided by magnesium stored in the intracellular compartment. About 120 mg of magnesium are eliminated into the urine every day contributing substantially to magnesium homeostasis [47]. Renal magnesium exchanges are closely dependent on magnesium body status, because magnesium depletion increases magnesium reabsorption. Thus, urinary excretion is reduced in magnesium-depleted conditions [48]. Diuretic, drugs that are commonly used in hypertension and heart failure, may as well modify renal magnesium exchanges by reducing the reabsorption of magnesium [49]. No known hormonal factor is specifically involved as a main regulator of magnesium homeostasis. Nevertheless, several hormones have been shown to exert actions on magnesium balance and transport, including parathyroid hormone, calcitonin, catecholamines, and insulin [3,50]. 

Table 1 depicts some food sources of magnesium, which correspond to foods belonging to dietary patterns generally considered healthy. Contrariwise, the foods contained in the Western diet, most of them ultra-processed, are very poor in magnesium. Ultra-processed food, according to NOVA classification, the most widely used classification of processed food, is defined as the “formulations of food substances often modified by chemical processes and then assembled into ready-to-consume hyper-palatable food and drink products using flavors, colors, emulsifiers and other cosmetic additives” [51]. In the last decades, the global supply of food products derived from industrial processes has increased substantially. The percentage of energy intake derived from ultra-processed foods has been reported to be 29.1% in France [52], 42% in Australia [53] and 57.9% in the USA [54]. Parallel to this transition towards diets based on processed food, a remarkable increase of non-communicable diseases, including obesity and hypertension, has been reported worldwide [55]. There is growing evidence linking consumption of this type of foods with poor diet quality, increased cardiovascular risk factors (e.g., hypertension, dyslipidemia), and harmful health outcomes such as obesity, metabolic syndrome [51] and also with increased mortality risk [52,56,57]. Negative nutritional characteristics of ultra-processed foods include its high content of low-quality fat, added salt and sugar, as well as low vitamin, mineral and fiber content [51].

Regarding the actual dietary sources of magnesium, in the USA, where 57.9% of energy intake comes from ultra-processed food [54], a study analyzing data from the National Health and Nutrition Examination Survey (NHANES) 2003 to 2008 among 25,351 participants, found that minimally processed food contributed only 27.6% to total magnesium intake, whereas ready-to-eat foods and packaged ready-to-eat foods contributed 28.8% and 26.3% to dietary magnesium intake, respectively [58]. This corresponds with the low consumption of foods rich in magnesium reported by the Dietary Guidelines for Americans (estimated % of persons below recommendation across all ages and both sexes in the USA was near 100% for whole grains, near 90% for total vegetables, over 80% for beans and peas, and near 60% for nuts, according to data from NHANES 2007–2010), which also indicated that 49% of the USA population, considering all age-groups, had a magnesium intake below the estimated average requirement [32]. Other estimates indicated that over 60% of Americans are under the recommended daily intake [28].

Data from the European Prospective Investigation into Cancer and Nutrition (EPIC) study reported that in Nordic and central European countries (i.e., Germany, UK, the Netherlands, Denmark, Sweden, and Norway), a large proportion (76 to 79%) of magnesium intake comes from highly processed foods. In Southern European countries, (i.e., Italy, Spain, and Greece) a lower proportion of magnesium intake derives from highly processed foods (43 to 67%), which is lower than in Nordic and central European countries, but still high [59]. Because highly processed foods are in general poor in magnesium, this may mean that people, both from the USA and European countries, consume large amounts of this type of foods to obtain magnesium, which is in any case insufficient, as indicated by the finding of frequent dietary magnesium deficiency at the population level [26,27,28,29].

The main sources of dietary magnesium, some examples shown in Table 1, contain also other components known to have beneficial health effects, i.e., other minerals and micronutrients, vitamins, fiber, and phytochemicals with recognized antioxidant and anti-inflammatory actions. Therefore, magnesium intake may be a marker of adherence to a healthy diet at a population level. Analyses of data from the Seguimiento Universidad de Navarra (SUN) prospective project showed that a higher adherence to the Mediterranean dietary pattern was associated with a lower prevalence of inadequacy for the intake of vitamins and minerals, including magnesium. Conversely, participants with a higher adherence to the Western dietary pattern (with greater consumption of red and processed meat, eggs, sauces, precooked food, fast-food, energy soft drinks, sweets, whole dairy and potatoes) were less likely to achieve adequate intakes of vitamins and minerals, including magnesium. Participants in the fifth quintile of adherence to Western dietary pattern had a 2.5-fold increased risk for having more than ten nutrient intake recommendations unmet, comprising magnesium, when compared to the first quintile of adherence to Western dietary pattern [60].

## 3. Mechanistic Insights on the Relationship of Magnesium and Hypertension

Several mechanisms can help explain the connection between magnesium and high blood pressure, including its calcium antagonist actions and its effects on endothelial function, vascular tone, reactivity, vascular cells growth, vascular calcification, oxidative stress and chronic inflammation, and glucose metabolism, as will be discussed in the below subsections (Table 2).

### 3.1. Regulation of Vascular Tone and Contraction

Magnesium is a major physiological regulator of vascular tone, and modulates peripheral vascular resistance by enhancing relaxation responses and mitigating agonist-induced vasoconstriction. The effects of magnesium as a modulator of vascular tone are also connected to its competitive action with calcium, while other mechanisms may be also involved as discussed below.

#### 3.1.1. Magnesium as a Calcium Antagonist

Calcium ion plays a crucial role in the control of vascular smooth muscle cells excitation, contraction and impulse propagation. All modifications of the endogenous magnesium status determine changes in vascular tone and, consequently, variations in blood pressure [4,8]. Although magnesium is not directly involved in the contraction process, it plays a role in blood pressure regulation through modulation of vascular smooth muscle tone and contractility by controlling calcium ion concentrations and availability [61,62]. Thus, a reduction of magnesium levels raises smooth muscle calcium content; while on the contrary, an increase of magnesium concentrations reciprocally lowers calcium content in the cells [63,64]. Extracellular magnesium levels and cellular-free magnesium concentrations modulate vascular smooth muscle cells tone by voltage-dependent L-type calcium channels [64]. Furthermore, magnesium can itself function as a natural physiologic calcium channel blocker [65], modulating the activity of the calcium-channels [66]. Thus, magnesium counteracts calcium and functions as physiological calcium blocker, similarly to synthetic calcium antagonists [67].

Magnesium binds hydration water more than calcium. Hence, the hydrated magnesium, with a radius of about four hundred times larger than its radius after dehydration, is more challenging to dehydrate. This dissimilarity clarifies many of magnesium biological properties, including its calcium antagonistic actions, in spite of similar chemical charge and reactivity of both ions. As such, it is almost impossible for magnesium to pass through narrow channels in biological membranes, opposite to calcium, because of its hydration cover [68].

Two mechanisms are proposed for the extracellular magnesium-inhibition of calcium current in vascular smooth muscle cells. On one hand, extracellular magnesium would stabilize the excitable membranes and raise the excitation threshold which diminishes the current via the voltage-gated calcium channels by neutralizing the negative charges on the external surface of the cell membrane. On the other hand, it has been suggested that extracellular magnesium may reduce calcium current by directly binding to the calcium channels. Magnesium may either cause an allosteric modulation of the channel gating, or mechanically block the channel pore, thus causing its closure [69].

In vascular smooth muscle cells, the concentration of intracellular magnesium modulates their tone by means of its effects on ion channels and calcium signal transduction pathways. As mentioned, decreased extracellular magnesium activates calcium influx, while raised extracellular magnesium levels inhibit calcium influx through calcium channels [64]. Variations in intracellular magnesium modulates channels activity by altering its amplitude, activation/inactivation kinetics, and by factors such as phosphorylation, thus reducing calcium entry. The magnesium-related activation of the calcium-ATPase pump in the sarcoplasmic/endoplasmic reticulum sequesters intracellular calcium into the sarcoplasmic reticulum. Elevated intracellular magnesium stimulates inositol-1,4,5-trisphosphate (IP3) breakdown, inhibits IP3-induced calcium release from the sarcoplasmic reticulum, and competes with intracellular calcium for cytoplasmic and reticular binding sites [69]. Contrariwise, low concentrations of intracellular magnesium stimulate IP3-mediated mobilization of calcium from the sarcoplasmic reticulum and reduce calcium-ATPase activity, reducing calcium efflux and reuptake by the sarcoplasmic reticulum. This causes an accumulation of cytosolic calcium and a raised cellular calcium concentration, which is a crucial factor for vasoconstriction [64]. Magnesium can also block sarcoplasmic reticulum calcium release through the ryanodine receptor [70]. The action of magnesium to compete with calcium for binding sites on troponin C also modulates the activity of contractile proteins and their dynamics [71]. In addition, intracellular magnesium regulates vascular smooth muscle cells G-protein-coupled activity of various receptors, including those for angiotensin II (type 1), endothelin-1, vasopressin, and norepinephrine and epinephrine, as well as intracellular calcium signal transduction pathways, such as translocation of phospholipase C and activation of protein kinase C [69].

Considering all those previously described direct and indirect actions of magnesium on the vascular smooth muscle cells, it is plausible to propose a role of magnesium deficiency in the pathophysiology of alterations of blood pressure homeostasis, such as hypertension. Thus, elevation of blood pressure and vascular hyperreactivity can be induced in experimental models by diminishing magnesium both in the in vitro environment, or depleting magnesium in experimental animals [72].

#### 3.1.2. Magnesium and Endothelial Function

Magnesium stimulates vascular endothelial functions by affecting the release of nitric oxide, endothelin-1, and prostacyclin [73]. Magnesium ions directly trigger the production of prostacyclin and nitric oxide [74,75] and its concentrations were found inversely related to endothelin-1 in hypertension experimental models [76], further supporting the ability of magnesium to modulate vasodilatation. Magnesium deficit have been shown to potentiate endothelial dysfunction by means of the activation of nuclear factor kappa-light-chain-enhancer of activated B cells (NF-kB), a well-known transcription mediator of proinflammatory pathways [77], Low concentrations of extracellular magnesium reduces endothelial cell proliferation, stimulates monocytes adhesion, and impairs vasoactive molecules, such as nitric oxide and prostacycline [78]. Another key mediator of magnesium’s effects on the endothelium is interleukin (IL)-1alpha, regulated by NF-kB, which in turn may be an inducer of NF-kB. IL-1alpha increases sharply in a low magnesium content environment and induces the production of various chemokines and adhesion molecules in vascular endothelial cells by activating NF-kB, hence, provoking adhesion, aggregation, and diapedesis of monocytes. Reduced magnesium concentrations trigger the secretion of IL-8 and chemokines overexpressed in human atherosclerotic plaques, promoting monocyte adhesion and chemotaxis to endothelial cells. IL-8 also stimulates proliferation and migration of vascular smooth muscle cells. The secretion of IL-1alpha induced by low serum magnesium stimulates overexpression of vascular cell adhesion molecule (VCAM)-1 on the surface of endothelial cells, which contributes to leukocyte migration. Granulocyte-macrophage colony-stimulating factor is also significantly higher in endothelial cells with magnesium deficit [78].

Supporting the key role of magnesium on endothelial function, oral magnesium supplementation was significantly associated with improvement in exercise tolerance and brachial artery endothelial function, in patients with coronary artery disease [11]. Likewise, oral magnesium improved endothelial function in type 2 diabetic older adults evaluated by non-invasive flow-mediated dilatation of the brachial artery [10]. A recent systematic review and meta-analysis summarized the effects of oral magnesium supplementation on vascular function in RCTs. Even if available studies were scarce and heterogeneity was high among the studies included, in subgroup analyses oral magnesium significantly improved flow-mediated dilation in studies longer than 6 months, including unhealthy persons, older than 50 years, or with BMI higher than 25 kg/m^2^ [79].

#### 3.1.3. Magnesium and the Renin-Angiotensin-Aldosterone System (RAAS)

In 1983, a study by Resnick et al. evaluated the relationship between plasma renin activity and serum concentrations of ionized calcium and magnesium in normotensive and hypertensive patients clustered into low-renin, normal-renin, and high-renin groups. Overall, the range of plasma renin activity in hypertensive participants showed a continuous and close inverse relation with serum ionized magnesium concentrations and a positive relation with serum ionized calcium [6]. The authors concluded that plasma renin activity may reflect modifications in calcium and magnesium fluxes across cell membranes in hypertension.

In experimental models, it has been shown that magnesium has some direct effects on the synthesis of aldosterone and indirect effects through the RAAS [80]. Aldosterone secretion is a calcium-dependent process, which can be affected by magnesium due to its calcium antagonist properties mentioned above. Rats maintained in a magnesium-deficient diet exhibited a slight reduction of the thickness of the inner zones and an increment of the juxtaglomerular granulation index and width of the zona glomerulosa of the adrenal cortex. When magnesium was restored in the diet, the thickness of the zona glomerulosa returned to normal [81]. Infusion of magnesium in humans decreased the production of aldosterone induced by angiotensin II, and on the contrary, dietary-induced magnesium deficiency enhanced angiotensin-induced aldosterone synthesis [82]. Magnesium supplementation has been shown to improve the pressor effects of angiotensin II and stimulate the production of vasodilator prostacyclin [74,75].

#### 3.1.4. Magnesium and Catecholamines

The release of catecholamines from the adrenal gland and from adrenergic nerve terminals in response to sympathetic stimulation is a calcium-mediated process. As discussed above, magnesium competes with calcium for membrane channels, blocking the calcium entrance, and consequently modifying these calcium-linked responses. The ability of magnesium to prevent the release of catecholamines from both the adrenal gland and peripheral adrenergic nerve terminals was shown in earlier laboratory experiments [83]. Based on these effects, magnesium sulfate was used with benefit in patients with phaeochromocytoma undergoing surgery in order to help control cardiovascular changes at induction and tracheal intubation during anesthesia [84]. Subsequent cases confirmed the beneficial effects of magnesium sulfate on life-threatening pheochromocytoma crisis with hypertensive encephalopathy and catecholamine-induced cardiomyopathy [85]. Also in patients undergoing anesthesia for other reasons, pretreatment with magnesium sulfate attenuated the systolic blood pressure upsurge and the rise in norepinephrine and epinephrine after tracheal intubation [86]. Magnesium is needed for the catalytic action of adenylate cyclase. As such, in the absence of magnesium the decreased activity of adenylate cyclase leads to an increased secretion of acetylcholine from preganglionic nerves, which in turn triggers further release of catecholamines from the adrenal glands [87].

Experimental animals fed with a magnesium-deficient diet showed a significant rise in catecholamines excretion [88]. Also in an experimental model of hypertension, it has been reported that magnesium has important sympatholytic effects by blocking N-type calcium channels at nerve endings, inhibiting norepinephrine release, and decreasing blood pressure independently of its direct vasodilating actions [89]. These effects are very relevant considering that sympathetic stimulation plays a pivotal role in the regulation of arterial blood pressure [83]. A recent systematic review and meta-analysis evaluating the effectiveness of intravenous magnesium sulfate on the hemodynamic fluctuations associated with the creation of pneumoperitoneum in adults undergoing laparoscopic surgery showed a consistent reduction in the magnesium treated groups compared to placebo in heart rate, systolic, diastolic and mean blood pressures, at 5 min, 10 to 15 min, and 30 min after pneumoperitoneum, confirming its ability to blunt the physiologic sympathetic response associated with exposure to injurious stimuli [90].

#### 3.1.5. Magnesium and Vascular Calcification

Vascular calcification refers to the deposit of calcium in the arterial wall and is closely linked to high blood pressure. Hypertension is a risk factor for atherosclerosis and intimal calcification. Nevertheless, not all vascular calcifications take place with atherosclerosis, while calcification of the vessel media is associated with reduced elasticity and arterial stiffness, a major cause of isolated systolic hypertension particularly frequent in old age. Notably, vascular calcification, independent of its anatomical site, is itself a risk factor for cardiovascular mortality [91]. Some studies have indicated a protective effect of magnesium against vascular calcification, attributable to its calcium antagonistic effects including hydroxyapatite formation and calcium transport into the cells [92,93]. The possible mechanism to explain such protective effect has not been yet fully clarified. In experimental models, it has been reported that calcium deposition in the rat aortic wall dramatically increased when the magnesium concentration was increased considering also calcium concentration (ratio of magnesium:calcium = 1:1) compared to low magnesium concentration and high calcium concentration (ratio magnesium:calcium = 1:3), suggesting that the impact of magnesium on vascular calcification might be studied in association with calcium levels [9].

In primary human aortic vascular smooth muscle cells, increasing magnesium concentrations improved cell viability and normalized the release of proteins involved in vascular calcification [94]. In this in vitro experimental model, the formation of calcium–phosphate–apatite crystals assessed with a qualitative analysis suggested a potential beneficial effect of magnesium in reducing the number and intensities of crystal formation. The authors suggested that their results seem to exclude a physicochemical role of magnesium in altering crystal growth, composition or structure, but that this attenuating effect should be linked to an active cellular role [67]. Also in bovine vascular smooth muscle cells higher magnesium concentrations prevented calcification and inhibited the expression of osteogenic proteins, apoptosis and further progression of already established calcification [95]. One of the intracellular mechanisms identified as possible mediator of magnesium’s anti-calcifying effect is the inhibition of the Wnt/beta-catenin signaling pathway [96].

In community-dwelling participants of the Framingham Heart Study without any cardiovascular disease at baseline, self-reported dietary and supplemental magnesium intake was inversely associated with coronary and abdominal artery calcification [97], supporting a protective role of magnesium on vascular calcification and derived complications, such as isolated systolic hypertension, stroke and coronary heart disease events.

### 3.2. Magnesium, Insulin Action, Diabetes, and Cardiometabolic Syndrome

Hypertension is common among patients with diabetes, and it is a strong risk factor for atherosclerotic cardio-vascular disease, heart failure, and microvascular complications in diabetic patients [98]. A recent analysis of the tendency of diabetics to develop hypertension and of hypertensives to develop diabetes concluded that the development of diabetes and hypertension track each other over time and that a reduced insulin sensitivity is a common feature of both pre-diabetes and pre-hypertension and an index of progression to the two conditions [99]. The constellation of risk factors known as metabolic syndrome including hypertension, obesity, and impaired glucose tolerance/insulin resistance has compelling evidence of its association with magnesium deficiency [3,39,50,100,101,102,103,104]. Cardiometabolic syndrome represents a strong risk factor for cardiovascular events and for the progression to type 2 diabetes. There is also convincing evidence of the link between magnesium deficit and diabetes. Type 2 diabetes has been associated with both intracellular and extracellular magnesium depletion, mostly in patients with poorly controlled glycemic profiles, longer duration of the disease, and in those with macro- and microvascular chronic complications [50,105,106,107]. Reduction in intracellular and/or ionized plasma magnesium has been reported in diabetic patients with normal values of total magnesium [108,109,110].

One of the key mechanisms that may induce magnesium depletion in diabetes is a low dietary magnesium intake and an increase in magnesium urinary loss, while absorption and retention of dietary magnesium appears to be unmodified in these patients [111]. A diet deficient in magnesium, very common in western dietary patterns full of ultra-processed food, has been associated with an impaired cellular insulin-mediated glucose uptake and with a remarkably high risk of developing glucose intolerance and type 2 diabetes [50]. On the other hand, magnesium depletion in diabetic patients has been related to renal calcium and magnesium wasting. It has been suggested that both, hyperglycemia and hyperinsulinemia, may play a role in the increased urinary magnesium excretion contributing to magnesium reduction. Urinary magnesium excretion rates were more than doubled in diabetic patients during hyperglycemia, in parallel with a reduction in plasma magnesium [112]. An effective metabolic control is associated with a reduced urinary magnesium wasting [107]. In addition, hyperinsulinemia, associated with insulin resistant conditions, may contribute per se to the urinary magnesium depletion, while reduced insulin sensitivity may itself affect magnesium transport [113]. In this way, lower magnesium levels may not only be a consequence, but may also predispose to the development of diabetes. Insulin resistance reduces renal magnesium reabsorption leading to urinary magnesium wasting. Thus, persons with type 2 diabetes may end up in a vicious circle in which hypomagnesemia causes insulin resistance and insulin resistance reinforces magnesium depletion [50,105].

After the introduction of insulin-containing extracts from animal pancreas as a lifesaving therapy for diabetes in the early 1920s [114], a study published in 1933 reported increased blood magnesium and sodium concentrations during therapy with impure insulin extracts [115]. Only 30 years later in 1960 when synthetic insulin was available and methods of magnesium measurements improved, it became apparent that insulin regulates magnesium renal reabsorption [116]. Afterwards, microperfusion experiments in mouse thick ascending limb of Henle loop showed an increased magnesium permeability after addition of insulin [117]. Furthermore, insulin stimulated magnesium uptake in mouse distal convoluted tubule cells [118]. It seems then clear that magnesium transport is a key molecular target to help explain the actions of insulin in the kidney.

In the last decades there have been advances in the study of magnesium transport systems, but the results of the available studies are still inconsistent. For example, in 2012, transient receptor potential melastatin type 6 (TRPM6) was identified as the molecular target of insulin signaling and some mutations in TRPM6 were proposed as responsible for rendering the channel insensitive to insulin stimulation in patch clamp analyses [119]. This was not confirmed when higher amounts of magnesium intake were examined together with possible genetic variations. Analyses of fifteen studies from the CHARGE (Cohorts for Heart and Aging Research in Genomic Epidemiology) Consortium providing data from 52,684 participants showed that magnesium intake was significantly and inversely associated with fasting glucose and insulin, after adjustment for age, sex, energy intake, body mass index, and behavioral risk factors. No magnesium-related SNP (single nucleotide polymorphism) or interaction between any SNP and magnesium reached significance after correction for multiple testing [120]. Also in experimental models, the mRNA expression of TRPM6 in diabetic rats, are contradictory with some reports showing increased TRPM6 expression [121], and others showing downregulation of TRPM6 [122]. These inconsistencies, as in other animal studies, may depend on the different experimental model used. Moreover, because hypomagnesemia may per se stimulate TRPM6 expression [123], it is difficult to isolate the effects of hypomagnesemia from those of diabetes itself.

Another transport system involved in the renal actions of insulin is the thiazide-sensitive Na-Cl cotransporter in the distal convoluted tubule [124,125,126,127]. Insulin stimulation of this system has been shown to increase sodium reabsorption by activating an intracellular signaling cascade that includes mTOR complex 2 and stress-activated protein kinase/oxidative stress responsive kinase to increase Na-Cl cotransporter phosphorylation and activity [124,126,127]. It is noteworthy that, as mentioned above, all phosphorylation reactions are magnesium dependent. It has been suggested that hyperinsulinemia in patients with diabetes may cause an increased activation of Na-Cl cotransporter, hence, of renal sodium reabsorption, contributing to hypertension that is so common in type 2 diabetic patients [98]. This assumption is backed by studies in Zucker obese rats and db/db mice showing hypertension, hyperinsulinemia, and increased Na-Cl cotransporter activity [125,126].

In epidemiological studies magnesium deficit has been linked to an increased risk of glucose intolerance, type 2 diabetes and cardio-metabolic syndrome [39,100,128]. Depletion of intracellular magnesium inducing an altered activity of the tyrosine kinase insulin receptor, as well as all other magnesium-dependent kinases of the insulin signaling, impairs insulin sensitivity and may contribute to the development of clinical conditions associated with a reduced insulin sensitivity, such as glucose intolerance, type 2 diabetes and hypertension. Additional mechanisms proposed to explain the link of magnesium with insulin resistance/metabolic syndrome are inflammation and oxidative stress. In general, conditions commonly associated with magnesium deficiency, such as diabetes and aging, are also associated with increased free radical formation and derived damage to cellular processes [50,129]. The view that a dietary magnesium deficit may cause and/or exacerbate insulin resistance is confirmed by data, both in experimental animals [130] and in humans [82], showing that a diet poor in magnesium is associated with insulin resistance. A magnesium-deficient diet caused a significant impairment of insulin-mediated glucose uptake in sheep [131], while magnesium supplementation delayed the development of diabetes in a rat model of diabetes [132]. A higher intake of magnesium was related to lower fasting insulin concentrations among non-diabetic women [133], and a significant inverse association was present between total dietary magnesium intake and the insulin responses to an oral glucose tolerance test [134]. Because of this reported increased risk for developing glucose intolerance and type 2 diabetes in persons with dietary magnesium deficits, it has been proposed a potential benefit of dietary magnesium supplementation, as a preventive tool in persons with diabetes or at risk for developing type 2 diabetes. However, the number of studies concerning magnesium supplementation in people with or at risk of diabetes is still limited [135]. Benefits of Mg supplementation on glucose control improvements have been suggested in most, but not all, studies. A systematic review and meta-analysis from our group including eighteen double-blind randomized controlled trials (RCTs), twelve in people with diabetes and six in people at high risk of diabetes, showed that magnesium supplementation appears to have a beneficial effect improving glucose parameters in persons with diabetes and also improving insulin-sensitivity parameters in those at high risk of diabetes [136].

### 3.3. Magnesium, Oxidative Stress and Chronic Inflammation

The etiology of hypertension involves the complex interaction among various elements, including genetic, environmental, anatomic, adaptive, neural, endocrine, humoral, and hemodynamic factors, first described by Irvine Page in his mosaic theory [137]. Since then, with the enormous progress in hypertension research it has become apparent that common molecular and cellular events in various organs lie beneath many features of the original mosaic theory. In 2013, David Harrison highlighted oxidative stress and inflammation as major drivers harmonizing diverse cellular events and organ systems involvement in hypertension, revisiting Page’s theory. Harrison proposed that oxidative stress and inflammation increase neuronal firing in specific brain centers, increase sympathetic outflow, alter vascular tone and morphology, and cause sodium retention in the kidney together with other cellular signals, including calcium signaling and endoplasmic reticulum stress [138]. The crucial role of inflammation in cardiovascular and metabolic disease was first proposed by Ross in the 1990s, showing that excessive inflammatory-fibroproliferative responses to various forms of injury to arterial endothelium and smooth muscle are soundly involved in atherogenesis [139]. Nowadays, there is compelling experimental and clinical evidence indicating that hypertension is associated with inflammation, fibrosis, and activation of immune cells, processes that are driven in large part by oxidative stress [140]. Expression of vascular cell adhesion molecules (VCAMs), production of inflammatory mediators (e.g., tumor necrosis factor [TNF], IL-1, IL-6, 1L-17), stimulation of proinflammatory signaling pathways (e.g., mitogen-activated protein kinase [MAPK], signal transducer and activator of transcription [STAT]), activation of transcription factors (e.g., NF-kB, STAT activator protein 1, hypoxia-inducible factor 1), and circulating levels of inflammatory biomarkers (e.g., C-reactive protein [CRP], plasminogen activator inhibitor [PAI]-1, ILs) are all increased in hypertension [141,142,143]. Although it still remains unclear whether inflammation is a cause or an effect of hypertension, it is clear that the immune system and oxidative stress are important players.

Along with all the above-mentioned actions on key mediators of hypertension, it has been convincingly shown that low blood concentrations of magnesium are associated with an increased production of oxygen free radicals also known as reactive oxygen species (ROS). Also diets with poor magnesium content have been linked to a low-grade chronic inflammatory state, mainly by two mechanisms: first, by initiating an excessive production and release of IL-1beta and TNF-alfa, and second, by triggering the synthesis of nitric oxide and of some inflammatory markers [144,145]. Magnesium deficiency increases platelet aggregation and adhesiveness, and inhibits growth and migration of endothelial cell, potentially altering microvascular functions [145].

Several studies in experimental models have shown that magnesium deficiency causes: (i) elevation of proinflammatory molecules TNF-alfa, IL-1-beta, IL-6, VCAM-1, and PAI-1 [145,146]; (ii) increased circulating inflammatory cells [147]; and (iii) increased hepatic production and release of acute phase proteins (i.e., complement, alfa2-macroglobulin, fibrinogen) [145,148]. As mentioned above, endothelial dysfunction associated with low magnesium exposure has been linked to the release of inflammatory mediators [149].

In humans, clinical data have demonstrated that reduced serum magnesium levels as well as low dietary magnesium intakes are strongly associated with low-grade systemic inflammation [12,28,150]. Other studies have shown an inverse relationship of dietary magnesium intake and serum magnesium with inflammation markers. The Women’s Health Study has shown that magnesium dietary intake was inversely related to systemic inflammation, measured by serum CRP concentrations, as well as with the prevalence of the metabolic syndrome in adult women [103]. Magnesium intake was inversely longitudinally related to incident diabetes in a large population of American adults, at least in part explained by the inverse association of magnesium intake with systemic inflammation and insulin resistance [151]. In addition, using the 1999–2002 NHANES databases, it was found that magnesium intake was inversely associated with CRP levels. Among 70% of the population studied, not taking magnesium supplements, dietary magnesium intake below the RDA was significantly related to an increased risk of having elevated CRP [28]. A recent investigation confirmed the significantly inverse relationship of low dietary magnesium intake with serum hs-CRP concentrations in a large Finish population [152].

Magnesium deficits have also been associated with decreased antioxidant defense competence and increased oxidative stress. There is evidence showing that magnesium depletion may cause an increased production of oxygen-derived free radicals in different tissues, decreased antioxidant enzyme expression and activity, decreased cellular and tissue antioxidant levels, increased production of superoxide anion by inflammatory cells and increased oxygen peroxide production and increased oxidative tissue damage [145,153].

Low serum magnesium (i.e., extracellular) can trigger magnesium transporters such as TRPM7 and solute carrier family 41 A1 (SLC41A1), a mammalian magnesium carrier [154], inducing magnesium efflux from cells to increase serum magnesium concentrations. This may decrease intracellular magnesium altering magnesium- and ATP-dependent cellular signaling functions. A decreased intracellular magnesium may trigger magnesium stores in the mitochondria to release magnesium [155] through SLC41A3 [156]. Reduced mitochondrial magnesium content may further compromise magnesium- and ATP-associated mitochondrial signaling and functions. This may explain the mitochondrial overproduction of ROS and decreased ATP observed in magnesium deficient mice [157,158]. Recently, it has been shown that magnesium deficiency in diabetic mice increased mitochondrial oxidative stress and contributed to cardiac diastolic dysfunction, which was reversed by magnesium supplementation [157]. Thus, magnesium can act as a mitochondrial antioxidant. Magnesium deficiency has been shown to alter mitochondrial function by several mechanisms, including alterations in coupled respiration [159,160,161], increasing mitochondrial ROS production [157,158,162], suppressing the antioxidant defense system (e.g., superoxide dismutase, glutathione, catalase, vitamin E) [163,164,165,166], inducing calcium overload via the mitochondrial calcium uniporter [157,167,168], attenuating pro-survival signaling [169,170,171], and promoting opening of mitochondrial ATP-sensitive potassium channel [172], inner membrane anion channel [173], and mitochondrial permeability transition pore [174]. These effects result in depolarization of the mitochondrial membrane potential [167]. Conversely, magnesium repletion has been shown to improve mitochondrial function by suppression of mitochondrial ROS overproduction [157,158], inhibition of mitochondrial permeability transition pore opening and cytochrome C release [175,176,177], preservation of mitochondrial membrane potential [178,179], reduction of mitochondrial calcium accumulation [180,181,182], increase of protein expression of the anti-apoptotic B-cell lymphoma 2 (Bcl-2) family and concurrently decreasing pro-apoptotic protein expression such as Bcl-2-associated X protein [169,179], decrease of apoptosis by suppressing activation of hypoxia-inducible factor 1alpha and p38 mitogenactivated protein kinase/c-Jun N-terminal kinase (p38/JNK) signaling [179], and by downregulation of autophagy [182].

We have previously proposed a link between the action of magnesium to alter the antioxidant capacity and to increase oxidative stress, inflammation, and lipid oxidation with the possible development of insulin resistance, type 2 diabetes, hypertension and cardio-metabolic syndrome [50]. Aging, very frequently associated with cardiovascular disease including hypertension, as well as with other chronic diseases, is characterized by a chronic, low-grade inflammatory state that involves several tissues and organs, and that has been named “inflammaging” [183]. Our group has suggested a link between the magnesium deficit through its role in causing a pro-oxidant –pro-inflammatory state to several age-related diseases and the low-grade inflammation associated with aging [15,129]. Magnesium itself has antioxidant properties scavenging oxygen radicals possibly by affecting the rate of spontaneous dismutation of the superoxide ion [184] and all the other mechanisms described above.

## 4. Hypertension in Old Age and Magnesium Deficit—Two Frequent Coexisting Conditions

Aging is accompanied by significant hemodynamic changes, leading to an ever-growing pandemic of hypertension. Modifications in central arterial structures are characterized initially by a decline in aortic distensibility with an increased diastolic blood pressure, followed by a sharp increase in pulse wave velocity (PWV), pulse pressure (PP) and systolic blood pressure, beyond the sixth decade. These trajectories of PWV and PP differ with advancing age. In addition, there is an increased prevalence of salt-sensitive hypertension in old age [185]. Epidemiological data from the Framingham Study suggest that the lifetime risk of incident hypertension is over 90% for a person aged 55 to 65 years [186]. Arterial stiffness is the major cause of elevated systolic blood pressure and PP (systolic minus diastolic blood pressure) as well as lower diastolic blood pressure in older adults. These age-related vascular alterations are powerful determinants of major cardiovascular disease events and all-cause mortality [187,188,189,190,191]. Vascular aging entails modifications in the properties of all the elements of the vascular wall, including endothelium, vascular smooth muscle, and extracellular matrix, leading to vascular stiffness and possible elevation of systolic blood pressure. These age-related arterial changes and those associated with hypertension (and with diabetes and atherosclerosis) are strictly connected at the cellular and molecular levels [192]. In the young adult, arterial vessels adapt blood flow and pressure during cardiac systole to facilitate perfusion to tissues during diastole. This is largely determined by elasticity, distensibility, and compliance of the arterial wall. Increased stiffness and loss of elasticity need greater force to accommodate blood flow, leading to increased systolic blood pressure and consequent increased cardiac work load. Various interrelating factors at the systemic (blood pressure, hemodynamics), vascular (vascular contraction/dilatation, extracellular matrix remodeling), cellular (cytoskeletal organization and inflammatory responses in endothelial cells and vascular smooth muscle), and molecular (oxidative stress, intracellular signaling, and mechanotransduction) levels contribute to arterial stiffness in hypertension [187,188,189]. Modifications in magnesium status and cellular content play a key role in many of these processes as discussed in previous subsections. Hence, interventions focused on correcting magnesium deficiency and maintaining an optimal magnesium balance may prove to be an appropriate strategy against arterial aging due to its positive effects on various mediators of the vascular aging process.

In experimental models, the effects of magnesium deficit and supplementation on the mechanical properties of common carotid artery were assessed continuously with an echo-tracking device. Histological examination showed a larger cross-sectional area, increased intima-media thickness and a greater media:lumen value in carotid artery of magnesium-deficient rats, suggesting growth and/or proliferation of arterial wall components in this condition. A negative linear relationship between intima-media thickness and plasma magnesium concentration was reported [193]. Another experimental study compared young and old rats with long-term magnesium-deficient diet vs. magnesium-supplemented diet. Old rats fed a normal diet (not deficient or supplemented) showed increased PP, increased aortic wall thickness, loss of endothelium-dependent relaxation, and a decrease of the aortic wall elastin/collagen ratio. Long-term magnesium deficiency progressively increased systolic blood pressure and intra-arterial PP. Histological examination showed that magnesium deficiency increased the age-induced deleterious effects on composition and structure of aorta (media thickness, increased collagen content and reduction in the elastin/collagen ratio), which led to large artery rigidity [194]. In humans, aortic distensibility measured with MRI imaging in the descending thoracic and abdominal aorta in relation to ^31^P-MR spectroscopic measurement of in situ intracellular free magnesium levels in brain and skeletal muscle showed that aortic distensibility in hypertensive patients was consistently and significantly reduced as was brain and muscle intracellular magnesium, while systolic blood pressure was inversely related to aortic distensibility [13]. Another frequent characteristic of hypertension associated with aging is sodium sensitivity [195]. It has been shown that the ability of a high salt diet to elevate blood pressure is related to intracellular free magnesium in humans [196].

Along with the higher prevalence of hypertension, especially systolic due to arterial stiffness, aging is frequently associated with magnesium deficiency [129]. The total body and intracellular magnesium content tend to decrease with age. Aging is often associated with magnesium deficiency due to reduce intake and/or absorption, increased renal wasting and/or reduced tubular reabsorption, as well as age-related diseases and their treatment with certain pharmacological therapies [129]. In general, total plasma magnesium concentrations do not change with age [197]. Variability in magnesium circulating concentrations is generally associated with the presence of age-related diseases and modifications in renal function. An increased magnesium retention rate has been shown in old age, suggesting a significant subclinical magnesium deficit, not detected by the usual measurements of total serum magnesium [198]. We observed a decline in intracellular free magnesium with age; specifically, we studied the trend of intracellular magnesium content with age, using the gold standard method (^31^P-NMR spectroscopy) in healthy young and older persons and observed a continuous age-dependent fall of intracellular magnesium levels in red blood cells of healthy older adults [199], while total serum magnesium was not modified in the different age groups. Many older adults are susceptible to chronic latent magnesium deficiency and epidemiological data from the US and Europe have confirmed that low magnesium intake is very common [26,27,28,29,200], in societies in which it is usual that processed and ultra-processed foods are the basis of the diet [51,53,54]. This type of dietary pattern is very poor in components of high nutritional value, that is, essential macro and micronutrients including magnesium [30].

Malnutrition is a common geriatric syndrome, frequently connected to frailty [201,202], particularly in very old persons. A multicenter study from Ireland showed that 63% of persons aged over 70 years were malnourished or at risk for malnutrition [203]. Another multicenter study including 4500 older adults from twelve European countries in diverse geriatric settings reported that two-thirds of participants were at risk of malnutrition or malnourished [204]. Numerous factors contribute to malnutrition in old age including decreased appetite due to reduced sense of smell and taste, poor oral health, loss of vision and hearing, and depression-associated anorexia; decreased ability to purchase and prepare food, altered energy need, decreased physical activity and sarcopenia, loss of self-sufficiency, isolation, and financial limited access to food [201]. All these factors may certainly result in poor diets lacking essential nutrients including magnesium. A former study showed that magnesium intake in older persons was near half of recommended dietary allowance (RDA) [205]. Other studies confirmed the fact that older populations have low dietary intake of magnesium [206,207,208]. Perhaps older adults are more likely to experience low magnesium intake for the reasons described above, but indeed, this is a problem in the whole population regardless of age [209]. The RDA of magnesium in the US is 420 mg/day for men and 320 mg/day for women, requirement that do not seem to change with age [210], but the mean intake of magnesium in the US older population is far below this recommendation (225 and 166 mg/day for men and women, respectively) [26]. Sixty-eight per cent of US adult population has been shown to consume less than the RDA of magnesium, 45% consume less than 75% of the RDA, and 19% consume less than 50% of the RDA [28]. The “Suppléments en Vitamines et Minéraux AntioXydants” (SU.VI.MAX) French study showed that 77% of women and 72% of men had dietary magnesium intakes lower than RDA; and 23% of women and 18% of men consumed less than two thirds of the RDA [200]. The problem of dietary magnesium deficiency is even worse in nursing home residents [211,212,213,214,215,216,217].

Data from the NHANES III showed that magnesium intake tend to decrease with age [26]. Additionally, older people who suffer from chronic diseases and who use multiple medications have a higher risk of magnesium deficiency [15]. Decreased intestinal magnesium absorption may further contribute to its deficiency in old age [218]. Magnesium absorption occurs mainly in the duodenum and ileum by both passive and active transport. Alterations of magnesium intestinal absorption in old age may be worsen by the common age-related impaired vitamin D homeostasis [29]. Latent primary renal disorders frequent in older adults may also be associated with an increased magnesium loss linked to a reduced renal tubular reabsorption.

Secondary magnesium deficiencies may be associated with the use of multiple medicaments, known as polypharmacotherapy (i.e., loop diuretics, thiazides, proton pump inhibitors, cytotoxic drugs, digoxin, aminoglycosides, steroids), or with some pathological conditions (e.g., type 2 diabetes, insulin resistance, alcoholism, hyperadrenoglucocorticism, HIV/AIDS, acute myocardial infarction, stroke, etc.). One of the most frequently used drugs in the cure of hypertension are diuretics, which by increasing magnesium urinary loss can be a frequent cause of hypomagnesemia [219]. It has been reported the finding of hypomagnesemia in 38% to 42% of hypokalemic patients. The correction of potassium and/or calcium deficits may be difficult to achieve unless the magnesium deficit is also corrected, hence in patients with hypokalemia and/or hypocalcemia, a magnesium deficiency should be considered [220]. Unfortunately, there are no readily and easy methods to accurately assess magnesium status. The serum magnesium (only 1% of total body magnesium) is easily available but may not adequately reflect body magnesium stores which are mostly intracellular. Normal circulating concentrations may be found even if intracellular magnesium is depleted because intracellular stores are recruited to keep serum concentrations within normal range [3,110]. Therefore, as no fully accurate and robust method to measure magnesium status is available, the biochemical measurements should always be supported by a clinical assessment of patients at risk for magnesium deficiency in order to timely star a proper therapy.

Many other medications may reduce magnesium absorption and/or diminish magnesium circulating concentrations (e.g., proton pump inhibitors, antacids, H2 blockers, antibiotics, antivirals, antiepileptic drugs, and antihistamines, among others) [220]. Hypomagnesemia can become severe when different factors are combined, such as those described in a case report of posterior reversible encephalopathy syndrome (PRES) with associated hypertension, and reversal of symptoms after normalization of magnesium blood levels by magnesium administration and suspension of a proton pump inhibitor [221].

Western diets are generally very low in green vegetables and whole grains (as those examples in Table 1), and rich in refined foods, and are often severely deficient in magnesium. Most of the magnesium present in processed food is lost in refining procedures, and thus, diets that provide a high proportion of daily calorie requirements from refined or processed foods are likely to be low in magnesium [222]. Magnesium deficiency in plants is becoming an increasingly severe problem linked to the development of industrial agriculture [223]. Moreover, some pesticide agents, commonly used in the crops, such as glyphosate, may chelate minerals including magnesium [224] further decreasing the content of magnesium in soil and in some crops. Organic food, from pesticide-free soils, has been reported to have significantly more magnesium than non-organic control food [225]. Table 3 summarizes the mechanisms of magnesium deficiency in old age.

## 5. Methods

**Figure 1:**
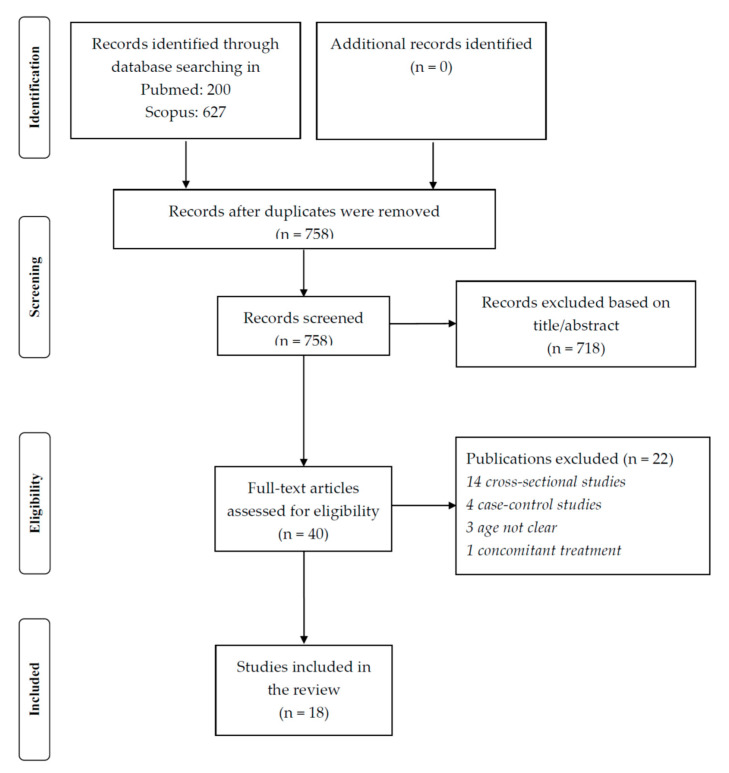
PRISMA flow-chart for the search and study selection.

We searched, from database inception to 16 December 2020, in Pubmed the topics of magnesium and hypertension using the following search for including all the studies (observational or interventional) eligible: “hypertension” [tiab] AND “magnesium” [tiab] Filters: Meta-Analysis, Observational Study, Randomized Controlled Trial, Systematic Review. A similar search was made in Scopus. Altogether, 200 title/abstracts were eligible from Pubmed and 627 from Scopus. After removing the duplicates, 758 title/abstracts were retrieved for a total of 40 works potentially eligible. Finally, a total of 18 eligible studies were considered for this narrative review (Figure 1 and Table 4).

Papers were considered eligible if: a. they included magnesium as treatment in placebo-controlled RCTs or if they assessed circulating or dietary magnesium in cohort studies, including meta-analyses of these works; b. they investigated hypertension as main condition in RCTs or as outcome in cohort studies. Concomitant supplementations (e.g., vitamin D), not clear definition of age or hypertension or cross-sectional/case–control design/no randomized controlled trials, studies made in children/adolescents/pregnant women were reasons of exclusion.

## 6. Available Evidence of the Effects of Dietary and Supplemental Magnesium on Hypertension

The role of magnesium as a therapy for hypertension in young and older adults, although first reported over 90 years ago for malignant hypertension and pre-eclamptic pregnancies [5], remains not completely defined. It is noteworthy that the use of intravenous magnesium sulfate in pregnancy-associated hypertension and especially in the prevention and treatment of seizures and PRES in eclampsia is well-established. This is based on evidence of beneficial effects in RCTs like the Magpie trial [229] and recommended in current guidelines [230,231,232], which prospects its validity in the third millennium after practically one hundred years of use. It should be noted that very high doses are used in pre-eclampsia and eclampsia and that the collateral effects are minimal considering that pregnant women are patients in whom particular caution is warranted due to the eventual consequences for both the mother and the newborn. Older hypertensive adults are frequently frail persons with multiple comorbidities who could potentially benefit from magnesium treatment.

Following the first use in 1925, magnesium continued to be utilized in hypertension associated with eclampsia, acute nephritis, and various vascular disorders, as testified by an article from 1942 including 40 cases with variable results [233], which discouraged further studies and recommendations of a regular use at that time. Results from different small trials remained non-homogeneous later on. Subsequent epidemiologic cross-sectional studies in the 1980′s and 1990′s suggested an inverse relationship between magnesium dietary intake and hypertension [33,34,35,234]. Straightforward recommendations were not possible upon cross-sectional studies, but the results suggested that foods rich in magnesium, such as vegetables, nuts, legumes, and whole grains may be protective against hypertension. The heterogeneous results of magnesium supplementation on the risk of hypertension, with some positive and some negative results, gave rise to a 2006 Cochrane review and meta-analysis suggesting that there was not yet enough information to recommend a wide use of magnesium in hypertension despite a small statistical reduction in diastolic blood pressure [226].

Currently, in addition to the former cross-sectional studies mentioned above [33,34,35,234], there is convincing evidence from prospective studies of an inverse relationship of dietary magnesium intake and of magnesium supplementation with the risk of incident hypertension [36,37,38,39,40], confirming a protective effect of the ion. There are few studies with non-optimal designs, two cross-sectional and one longitudinal, reporting negative or inconclusive results: one cross-sectional study from South Africa including a multiethnic heterogeneous population of 325 participants was inconclusive for a relationship between magnesium intake and blood pressure [42]; a longitudinal analysis of data from the Atherosclerosis Risk in Communities Study showed a significant inverse association of serum magnesium concentrations with incident hypertension in women that did not reach statistical significance in men (although the trend confirmed an inverse relationship), and no association between dietary magnesium intake and incident hypertension [41]; a cross-sectional analysis in two waves of data from the NHANES III and NHANES IV reported similar intakes of magnesium and other minerals in hypertensive and non-hypertensive participants in both surveys. However, the pattern of significantly lower mineral intake (potassium + calcium + magnesium) emerged as unique to persons with isolated systolic hypertension in both NHANES III and NHANES IV [43].

Three meta-analyses of RCTs found that participants receiving magnesium supplementation had a significant reduction in blood pressure values vs. controls [22,23,44]. The meta-analysis by Dibaba et al. included 11 RCTs and 543 participants followed up for periods ranging from one to six months. The daily dose of elemental magnesium used in the trials ranged from 365 to 450 mg. The pooled results indicated that magnesium supplementation had a significantly greater reduction in systolic and diastolic blood pressure when compared to controls without supplementation in patients with insulin resistance, prediabetes, or other non-communicable chronic diseases [22]. A second meta-analysis by Zhang et al. included 34 RCTs and 2028 participants with a median dose of magnesium supplementation of 368 mg/d for a median duration of 3 months. The authors reported a significant reduction in systolic and diastolic blood pressure, accompanied with an elevation of serum magnesium concentrations when compared to placebo. One month of therapy with 300 mg/d was sufficient to elevate serum magnesium and reduce blood pressure according to restricted cubic spline curve analyses. A greater reduction of blood pressure were found in trials with high quality or low dropout rate, but residual heterogeneity was also found when these factors were considered [23]. A third meta-analysis of RCTs by Verma et al. evaluating the effect of magnesium supplementation on cardiovascular risk factors (including hypertension) in diabetic and nondiabetic participants found a favorable effect of magnesium supplementation on systolic blood pressure, together with reductions in fasting plasma glucose, high-density lipoprotein and low-density lipoprotein cholesterol and triglycerides, effects that were stronger in diabetic participants with hypomagnesemia. The meta-analysis included 28 RCTs, but only four were conducted in hypertensive participants [44].

Former meta-analyses suggested benefit with less prominent but still positive effects, possibly due to heterogeneity of the studies included in the analyses [24,45]. A meta-analysis by Kass et al., included 22 RCTs and 1173 participants with a range of follow-up between 3 and 24 weeks, and a daily dose of elemental magnesium ranging from 120 to 973 (mean dose of 410 mg/d). Although not all trials showed a significant blood pressure reduction, combining them there was a significant decrease in systolic (minus 3–4 mm Hg) and diastolic (minus 2–3 mm Hg) blood pressure, which was stronger for trials with crossover designed and doses higher than 370 mg per day. Overall, the size of the effect increased in parallel with the dose increment. The authors concluded that magnesium supplementation had a small but significant reducing effect on blood pressure, which warranted the implementation of larger RCTs [24]. Another meta-analysis by Jee et al., including 20 RCTs, most of them very small, with a total 1220 participants and a daily dose of magnesium supplementation ranging from 241 to 964 mg (median dose 371.1 mg/d), resulted in a small but significant effect for systolic blood pressure; diastolic blood pressure was also reduced without reaching the statistical significance. Nevertheless, there was an apparent dose-dependent effect of magnesium on blood pressure with reductions of 4.3 mm Hg in systolic blood pressure and of 2.3 mm Hg in diastolic blood pressure for each 241 mg/day increment in magnesium dose. Because the trials included were heterogeneous, the authors suggested that adequately powered trials with sufficiently high doses of magnesium supplements were needed to confirm their results [45].

Another meta-analysis with a different design by Rosanoff et al., examining 44 studies that were sorted according to hypertension status, magnesium dose and anti-hypertensive medication usage, showed that some studies reported significant lowering of blood pressure with magnesium supplementation, while others did not. Therefore, they performed analyses of a uniform subset of seven studies from the original studies identified involving 135 hypertensive participants on anti-hypertensive medication continuously for at least six months, with no more than a two-week washout, and with a mean starting systolic blood pressure higher than 155 mm Hg. In this subset of studies, the authors showed significant blood pressure reductions with magnesium supplementation (mean reductions in mm Hg of 18.7 mm Hg for systolic blood pressure and 10.9 mm Hg for diastolic blood pressure). The rest of the original trials, not fulfilling the characteristics described above, showed heterogeneous results, probably including high- and low- or non-responder participants combined [227]. The authors argued that the modest results reporter in former meta-analyses by Kass et al. [24], Jee et al. [45], and the Cochrane review by Dickinson et al. [226], were probably due to the fact that they blended dissimilar studies, which contributed to underestimate the potential of magnesium in hypertension in some (but not all) participants. Un umbrella review of systematic reviews and meta-analyses of observational and interventions studies from our group found a high-class evidence for the association of diastolic blood pressure and magnesium in intervention studies with magnesium supplementation vs. placebo and moderate class evidence for systolic blood pressure. The evidence was suggestive for the association of a higher dietary magnesium intake with a lower risk of stroke in observational studies [31].

Regarding dietary magnesium intake, a systematic review and meta-analysis of cohort prospective studies assessed the association of dietary magnesium intake and serum magnesium with incident hypertension. The meta-analysis included nine studies (6 on dietary magnesium, 2 on serum magnesium, and 1 in both) of ten cohorts and 20,119 cases of hypertension in 180,566 participates. Results showed a significant inverse relationship between dietary magnesium intake and the risk of incident hypertension when comparing the highest intake group with the lowest. For each 100 mg/day increment in magnesium intake there was a 5% lower risk of incident hypertension. The relation between the serum magnesium levels and the risk of hypertension was only marginally significant [21]. Dietary patterns reported to significantly reduce blood pressure in hypertensive and pre-hypertensive patients include Dietary Approaches to Stop Hypertension (DASH) and Mediterranean diet [235], both rich in foods such as vegetables, nuts, whole cereals and legumes—optimal dietary sources of magnesium. Interestingly, DASH, ranked as the most effective dietary model in reducing blood pressure [235], emphasizes the high content of minerals, including magnesium.

Concerning blood magnesium levels, a meta-analysis of cohort studies evaluated the association of circulating magnesium concentrations with the incidence of coronary heart disease, hypertension, and type 2 diabetes, including 11 studies (3 with results on hypertension, 14,876 participants with 3149 cases and mean 6.7-year follow-up). The pooled relative risk of incident hypertension was 0.91 (95% confidence intervals 0.80, 1.02; NS) comparing the highest to the lowest category of circulating magnesium concentration. However, the trend was significant with every 0.1 mmol/L increment in circulating magnesium being associated with a 4% reduction in hypertension incidence [228]. A recent review examining meta-analyses on the effects of electrolytes on hypertension including 32 meta-analyses showed that magnesium had the greatest blood pressure lowering effect followed by potassium and by sodium/salt reduction [25]. Table 4 summarizes the results from prospective studies and meta-analyses of trials or cohort studies on the association of magnesium and hypertension included in the review.

In summary, almost all (five out of seven) prospective observational studies exploring the association of dietary magnesium intake with incident hypertension reported significant inverse associations, sometimes varying among men and women. Only one of these studies reported data on serum magnesium showing a significant inverse association of serum magnesium with incident hypertension in women but not in men. All seven meta-analyses on RCTs testing the effects of magnesium supplementation on blood pressure reported significant blood pressure lowering effects. A meta-analysis of observational studies evaluating the association of dietary magnesium with the risk of hypertension reported a significant inverse association with each 100 mg/d increment of magnesium intake being associated with a 5% reduction in hypertension risk. A meta-analysis of serum magnesium concentrations with the risk of hypertension reported marginally significant effects. A summary of meta-analyses and an umbrella review reported generally positive effects as well. Nevertheless, it should be taken into account that all meta-analyses indicated the presence of large heterogeneity among the hitherto available trials.

## 7. Conclusions

**Figure 2:**
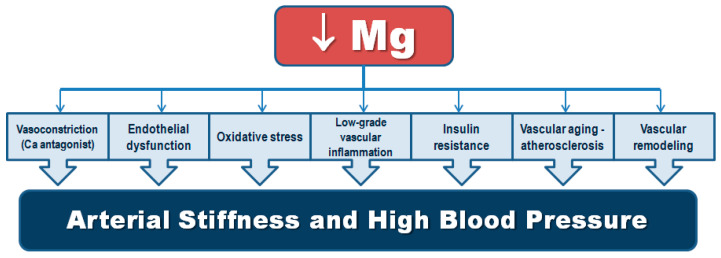
Combination of effects of magnesium by which magnesium deficit may lead to hypertension.

Over the past decades, there has been an outstanding amount of experimental, epidemiological, and clinical evidence showing a close relationship between magnesium deficit and high blood pressure. As shown in Figure 2, the multiple effects of magnesium on key mechanisms linked to the generation of arterial hypertension and its complications make this link strongly plausible and help to explain the bulk of evidence supporting a protective effect of magnesium against hypertension.

Magnesium has been used empirically for a century to treat severe hypertensive conditions, and in some cases, the most severe, such as pre-eclampsia, eclampsia-associated seizures and PRES, continues to be included in the guidelines of the third millennium, a century after having started its use. Hypertension is a complex condition in which various actors and mechanisms combine, resulting in cardiovascular and cerebrovascular complications that today represent the most frequent causes of mortality, morbidity, disability, and health expenses worldwide. This condition increases sharply with advancing age, hence older persons are those most affected by the negative consequences of hypertension. They are also more frequently at risk of magnesium deficiency by multiple mechanisms (Table 3), which may, at least in part, explain the higher frequency of hypertension and its long-term complications. Thus, older people have concurrently a higher risk for these two complex conditions. Moreover, the frequent use of diuretics as a therapy for hypertension and for one of its long-term complications, heart failure, can even worsen the magnesium deficiency.

Notwithstanding the convincing evidence that validates the possible key role of magnesium in hypertension, it is important to keep in mind that magnesium alone is not enough for hypertension prevention and/or treatment purposes. As mentioned, hypertension involves complex interactions among numerous endogenous and environmental factors; hence, the idea that it can be prevented or treated with a “magic bullet” is overstated and senseless. Furthermore, the main sources of dietary magnesium, i.e., vegetables, legumes, whole cereals, and nuts, contain also other components with health benefits, such as vitamins, other minerals and micronutrients, fiber, and phytochemicals with anti-inflammatory and antioxidant effects. At present, nutrition research emphasizes the impact of foods and nutrients combinations, as opposed to the reductionist approach based on single nutrients or foods, which was extensively considered in the past when diseases due to specific nutritional deficiencies were described and prevailing. Nevertheless, studies adjusted for multiple confounders have still reported independent associations of magnesium and hypertension. Moreover, at a population level, magnesium may be a marker of other significant risk factors for hypertension and of adherence to a healthy diet. Other non-dietary components of a healthy lifestyle are generally associated with a healthy diet [236]. Hence, dietary magnesium intake may be a marker of a healthy diet and lifestyle and may not only reflect the biological effect of an isolated healthy nutritional component.

The evidence for a beneficial effect of magnesium on hypertension risk emphasizes the importance of broadly encouraging the consumption of foods such as vegetables, nuts, whole cereals and legumes, optimal dietary sources of magnesium, avoiding processed foods, which are very poor in magnesium and lack other fundamental nutrients as well, in order to prevent hypertension. In some cases when diet is not enough to maintain an adequate magnesium status, magnesium supplementation may be of benefit and has been shown to be well tolerated.

## Figures and Tables

**Table 1:** Some Food Sources of Magnesium.

Food	Serving	Magnesium (mg)
Cereal all bran	½ cup	112
Cereal oat bran	½ cup dry	96
Brown rice, medium-grain, cooked	1 cup	86
Fish, mackerel, cooked	3 ounces	82
Spinach, frozen, chopped, cooked	½ cup	78
Almonds	1 ounce (23 almonds)	77
Swiss chard, chopped, cooked	½ cup	75
Lima beans, large, cooked	½ cup	63
Cereal, shredded wheat	2 biscuits	61
Peanuts	1 ounce	48
Molasses, blackstrap	1 tablespoon	48
Hazelnuts	1 ounce (21 hazelnuts)	46
Walnuts	1 ounce (14 walnuts)	44
Okra, frozen, cooked	½ cup	37
Milk, 1% fat	8 fluid ounces	34
Banana	1 medium	32

**Table 2:** Main Mechanisms of Magnesium-related Blood Pressure Regulation.

Regulation of vascular tone and contraction ○ Calcium antagonism ○ Endothelial function ○ RAAS ○ Catecholamine secretion ○ Vascular calcification Insulin resistance Oxidative stress and inflammation

RASS: Renin-Angiotensin-Aldosterone System.

**Table 3:** Main Mechanisms of Magnesium Deficit with Aging.

Primary magnesium deficit: ○ Inadequate magnesium dietary intake ○ Reduced efficiency of magnesium absorption (associated with reduced vitamin D levels)? ○ Increased urinary excretion of magnesium (associated with age-dependent reduction of kidney function and of magnesium tubular reabsorption) Secondary magnesium deficiency: ○ Associated with age-related diseases and comorbidities ○ Increased urinary magnesium loss secondary to drugs (i.e., diuretics) frequently used in hypertensive older adults

**Table 4:** Summary of results from prospective studies and meta-analyses of trials and cohort studies on the association of magnesium and hypertension included in the review.

Authors/Country	Year	N. of Trials or Prospective Cohort Studies	N. of Participants/Cases	Study Characteristics	Magnesium Dose	Duration of Follow-up or Trials	Summary of Results
Witteman et al. USA [36]	1989	-	58,218/3275	Prospective cohort	-	4 years	For women with high intakes of magnesium vs. low intakes, the RR of hypertension was 0.65 (95% CI, 0.53–0.80).
Ascherio et al. USA [38]	1992	-	30,681/1248	Prospective cohort	-	4 years	Among male health professionals, dietary magnesium was significantly associated with lower risk of hypertension after adjustment for age, relative weight, alcohol consumption, and energy intake.
Ascherio et al. USA [37]	1996	-	41,541/2526	Prospective cohort	-	4 years	Among women who did not report hypertension during follow-up, magnesium was significantly inversely associated with self-reported systolic and diastolic BP, after adjusting for age, BMI, alcohol consumption, and energy intake. Dietary magnesium was not significantly associated with risk of hypertension, after adjusting for age, BMI, alcohol, and energy intake.
Peacock et al. USA [41]	1999	-	7731/1577	Prospective cohort	-	6 years	Significant trend for the association of serum magnesium and incident hypertension in women, after adjustment for age, race, and other risk factors (*p* trend = 0.01) but not in men (*p* trend = 0.16). No association between dietary magnesium intake and incident hypertension.
Townsend et al. USA [43]	2005	-	10,033/1045 in NHANES III 2311/299 in NHANES IV	Two waves national survey	-		Similar intakes of magnesium and other minerals in hypertensive and non-hypertensive participants in both surveys. The pattern of significantly lower mineral intake (potassium + calcium + magnesium) emerged as unique to persons with isolated systolic hypertension in both waves.
He et al. USA [39]	2006	-	4637/608 MS	Prospective cohort	-	15 years	Magnesium intake was inversely associated with incidence of metabolic syndrome after adjustment for major lifestyle and dietary variables and baseline status of each component of the metabolic syndrome. The inverse associations were not modified by gender and race. Magnesium intake was also inversely related with individual component of the metabolic syndrome.
Song et al. USA [40]	2006	-	28,349/8544	Prospective cohort	-	9.8 years	Among women, magnesium intake was inversely associated with the risk of hypertension (*p* for trend < 0.0001 of magnesium quintiles). This inverse association was attenuated but remained significant after further adjustment for known risk factors (*p* for trend = 0.03). Similar associations were observed for women who never smoked and reported no history of high cholesterol or diabetes at baseline.
Jee et al. Korea, USA [45]	2002	20 (14 in hypertensives)	1220	Meta-analysis of interventional studies	10–40 mmol/d	3–24 wks	Apparent dose-dependent effect of magnesium on BP, with reductions of 4.3 mm Hg in systolic BP and of 2.3 mm Hg in diastolic BP for each 10 mmol/d increase in magnesium dose. Limiting the analysis to the 14 trials in hypertensives, for each 10 mmol/d of magnesium SBP was reduced by 3.3 mm Hg and DBP by 2.3 mm Hg.
Dickinson et al. UK [226]	2006	12	545	Cochrane review- Meta-analysis of RCTs	10–40 mmol/d	8–26 wks	On average, people receiving magnesium achieved slightly but significantly lower DBP (mean difference: −2.2 mmHg). Poor quality and heterogeneity of the trials. None of the studies reported any serious side effects.
Kass et al. UK [24]	2012	22	1173	Meta-analysis of interventional studies	120–973 mg/d	3–24 wks	Small but significant reduction in SBP of 3–4 mm Hg and DBP of 2–3 mm Hg, with greater increased in trials with crossover design and magnesium dose >370 mg/d.
Rosanoff et al. USA [227]	2013	7	135	Meta-analysis of interventional studies	10.5–18.5 mmol/d	6–17 wks	Significant mean reduction in SBP (mean −18.7 mmHg) and DBP (mean −10.9 mmHg) in hypertensives on continuous anti-hypertensive medication for at least six months, with no more than a two-week washout, and mean starting SPB > 155 mmHg.
Zhang et al. USA, China, Canada, Japan [23]	2016	34	2028	Meta-analysis of RCTs	238–960 mg/d	3 wks to 6 months	Significant reduction in SBP (mean −2.0 mmHg) and DBP (mean −1.78 mmHg) accompanied by 0.05 mmol/L rise in serum magnesium vs. placebo. Greater BP reduction found in trials with high quality or low dropout rate.
Dibaba et al. USA, Israel [22]	2017	11	543	Meta-analysis of RCTs	365–450 mg/d	1–6 months	Significant decrease in BP: mean reduction of 4.18 mm Hg in SBP and 2.27 mm Hg in DBP in participants with insulin resistance, prediabetes, or other noncommunicable chronic diseases.
Verma et al. India [44]	2017	28 (19 trials included for HTN analyses, 4 in hypertensives)	1694	Meta-analysis of RCTs	300–1006 mg/d	4–24 wks	Significant reduction in SBP (weighted mean difference = −3.056 mmHg) with greater beneficial effect in diabetic patients with hypomagnesaemia. High heterogeneity of the trials. In meta-regression, elemental magnesium dose was inversely DBP (*p* < 0.001).
Han et al. China, Sweden, USA, Norway [21]	2017	9	180,566/20,119	Meta-analysis of prospective cohort studies	-	4–15 years	Inverse association between dietary magnesium intake and the risk of hypertension. A 100 mg/d increment in magnesium intake was associated with a 5% reduction in the risk of hypertension. The association of serum magnesium concentration with the risk of hypertension was marginally significant.
Wu et al. China, USA [228]	2017	11 (3 on HTN)	Total: 38,808/4437 HTN: 14,876/3149	Meta-analysis of prospective cohort studies	-	6–8 years	Comparing highest vs. lowest category of circulating magnesium concentration, the pooled RR was 0.91 (95% CI 0.80, 1.02) for incident hypertension. Every 0.1 mmol/L increment in circulating magnesium levels was associated with 4% (RR 0.96; 95% CI: 0.94, 0.99) reduction in hypertension incidence.
Ikbal et al. Austria [25]	2019	8 (5 of RCTs, 3 of observational studies)	RCTs: 135–1694	Summary of meta-analyses	120–1006 mg/d	RCTs: 3–24 wks; observational studies: 4–15 years	The summary showed SBP reductions in the range of −0.2 and −18.7 mmHg, and DBP reductions between −0.3 and −10.9 mmHg. The meta-analysis [227] showing the largest effect, included a small sample of treated hypertensive patients, which probably responded highly to magnesium. When omitting this meta-analysis, the BP lowering effects of magnesium were attenuated to a low to moderate level. Observational studies showed a lower risk for hypertension with increasing magnesium intake or higher circulating magnesium levels.
Veronese et al. Italy, UK, Australia, Spain [31]	2020	16 meta-analyses	RCTs: 2262 participants in 34 RCTs; Observational studies: 180,566/20119	Umbrella review of systematic reviews and meta-analyses	120–1006 mg/d	RCTs: 3–24 wks; observational studies: 4–15 years	High class evidence for the association of diastolic blood pressure and magnesium in intervention studies with magnesium supplementation vs. placebo and moderate class evidence for systolic blood pressure. Large heterogeneity found for this outcome. The evidence was suggestive for the association of a higher dietary magnesium intake with a lower risk of stroke in observational studies.

BMI: body mass index; BP: blood pressure; CI: confidence interval; d: day; DBP: diastolic blood pressure; HTN: hypertension; MS: metabolic syndrome; RR: relative risk; SBP: systolic blood pressure; wks: weeks.

## Data Availability

No new data were created or analyzed in this study. Data sharing is not applicable to this article.

## References

[1] Ebel H., Günther T., Günther H.E.T. (1980). Magnesium metabolism: A review. Clin. Chem. Lab. Med..

[2] Caspi R., Altman T., Dreher K., Fulcher C.A., Subhraveti P., Keseler I.M., Kothari A., Krummenacker M., Latendresse M., Mueller L.A. (2011). The metacyc database of metabolic pathways and enzymes and the BioCyc collection of pathway/genome databases. Nucleic Acids Res..

[3] Barbagallo M., Dominguez L.J., Galioto A., Ferlisi A., Cani C., Malfa L., Pineo A., Busardo’ A., Paolisso G. (2003). Role of magnesium in insulin action, diabetes and cardio-metabolic syndrome X. Mol. Asp. Med..

[4] Gröber U., Schmidt J., Kisters K. (2015). Magnesium in prevention and therapy. Nutrients.

[5] Blackfan K., Hamilton B. (1925). Uremia in acute glomerular nephritis: The cause and treatment in children. Med. Surg. J..

[6] Resnick L.M., Laragh J.H., Sealey J.E., Alderman M.H. (1983). Divalent cations in essential hypertension. relations between serum ionized calcium, magnesium, and plasma renin activity. N. Engl. J. Med..

[7] Resnick L.M., Gupta R.K., Laragh J.H. (1984). Intracellular free magnesium in erythrocytes of essential hypertension: Relation to blood pressure and serum divalent cations. Proc. Natl. Acad. Sci. USA.

[8] Barbagallo M., Dominguez L.J., Resnick L.M. (2007). Magnesium metabolism in hypertension and type 2 diabetes mellitus. Am. J. Ther..

[9] Villa-Bellosta R. (2017). Impact of magnesium: Calcium ratio on calcification of the aortic wall. PLoS ONE.

[10] Barbagallo M., Dominguez L., Ligia J., Galioto A., Pineo A., Belvedere M. (2010). Oral magnesium supplementation improves vascular function in elderly diabetic patients. Magnes. Res..

[11] Shechter M., Sharir M., Labrador M.J.P., Forrester J., Silver B., Merz C.N.B. (2000). Oral magnesium therapy improves endothelial function in patients with coronary artery disease. Circulation.

[12] Song Y., Li T.Y., Van Dam R.M., Manson J.E., Hu F.B. (2007). Magnesium intake and plasma concentrations of markers of systemic inflammation and endothelial dysfunction in women. Am. J. Clin. Nutr..

[13] Resnick L.M., Militianu D., Cunnings A.J., Pipe J.G., Evelhoch J.L., Soulen R.L. (1997). Direct magnetic resonance determination of aortic distensibility in essential hypertension: Relation to age, abdominal visceral fat, and in situ intracellular free magnesium. Hypertension.

[14] Soave P., Conti G., Costa R., Arcangeli A. (2009). Magnesium and anaesthesia. Curr. Drug Targets.

[15] Barbagallo M., Belvedere M., Dominguez L.J. (2009). Magnesium homeostasis and aging. Magnes Res..

[16] King D.E., Mainous A.G., Geesey M.E., Ellis T. (2007). Magnesium intake and serum C-reactive protein levels in children. Magnes. Res..

[17] Benjamin E.J., Muntner P., Alonso A., Bittencourt M.S., Callaway C.W., Carson A.P., Chamberlain A.M., Chang A.R., Cheng S., Das S.R. (2019). Heart disease and stroke statistics—2019 update: A report from the American heart association. Circulation.

[18] Forouzanfar M.H., Liu P., Roth G.A., Ng M., Biryukov S., Marczak L., Alexander L., Estep K., Abate K.H., Akinyemiju T.F. (2017). Global burden of hypertension and systolic blood pressure of at Least 110 to 115 mm Hg, 1990–2015. JAMA.

[19] World Health Organization (2018). Global Health Observatory (GHO) Data. Blood Pressure. http://www.who.int/gho/ncd/risk_factors/blood_pressure_prevalence/en/.

[20] Olsen M.H., Angell S.Y., Asma S., Boutouyrie P., Burger D., Chirinos J.A., Damasceno A., Delles C., Gimenez-Roqueplo A.-P., Hering D. (2016). A call to action and a lifecourse strategy to address the global burden of raised blood pressure on current and future generations: The Lancet Commission on hypertension. Lancet.

[21] Han H., Fang X., Wei X., Liu Y., Jin Z., Chen Q., Fan Z., Aaseth J., Hiyoshi A., He J. (2017). Dose-response relationship between dietary magnesium intake, serum magnesium concentration and risk of hypertension: A systematic review and meta-analysis of prospective cohort studies. Nutr. J..

[22] Dibaba D.T., Xun P., Song Y., Rosanoff A., Shechter M., He K. (2017). The effect of magnesium supplementation on blood pressure in individuals with insulin resistance, prediabetes, or noncommunicable chronic diseases: A meta-analysis of randomized controlled trials. Am. J. Clin. Nutr..

[23] Zhang X., Li Y., Gobbo D., Liana C., Zhang W., Rosanoff A., Wang J., Song Y. (2016). Effects of magnesium supplementation on blood pressure: A meta-analysis of randomized double-blind placebo-controlled trials. Hypertension.

[24] Kass L.S., Weekes J., Carpenter L.W. (2012). Effect of magnesium supplementation on blood pressure: A meta-analysis. Eur. J. Clin. Nutr..

[25] Iqbal S., Klammer N., Ekmekcioglu C. (2019). The effect of electrolytes on blood pressure: A brief summary of meta-analyses. Nutrients.

[26] Ford E.S., Mokdad A.H. (2003). Dietary magnesium intake in a national sample of U.S. adults. J. Nutr..

[27] Mensink G.B.M., Fletcher R., Gurinovic M., Huybrechts I., Lafay L., Serra-Majem L., Szponar L., Tetens I., Verkaik-Kloosterman J., Baka A. (2013). Mapping low intake of micronutrients across Europe. Br. J. Nutr..

[28] King D.E., Mainous A.G., Geesey M.E., Woolson R.F. (2005). Dietary magnesium and C-reactive protein levels. J. Am. Coll. Nutr..

[29] Rosanoff A., Dai Q., Shapses S.A. (2016). Essential nutrient interactions: Does low or suboptimal magnesium status interact with vitamin D and/or calcium status?. Adv. Nutr..

[30] National Institutes of Health, Magnesium, National Institutes of Health, Bethesda, Maryland, USA, 2018. https://ods.od.nih.gov/factsheets/Magnesium-HealthProfessional/.

[31] Veronese N., Demurtas J., Pesolillo G., Celotto S., Barnini T., Calusi G., Caruso M.G., Notarnicola M., Reddavide R., Stubbs B. (2019). Magnesium and health outcomes: An umbrella review of systematic reviews and meta-analyses of observational and intervention studies. Eur. J. Nutr..

[32] Department of Health and Human Services (2020). US Department of Agriculture (2015) 2015–2020 Dietary Guidelines for Americans.

[33] Van Leer E.M., Seidell J.C., Kromhout D. (1995). Dietary calcium, potassium, magnesium and blood pressure in the Netherlands. Int. J. Epidemiol..

[34] Ma J., Folsom A.R., Melnick S.L., Eckfeldt J.H., Sharrett A., Nabulsi A.A., Hutchinson R.G., Metcalf P.A. (1995). Associations of serum and dietary magnesium with cardiovascular disease, hypertension, diabetes, insulin, and carotid arterial wall thickness: The aric study. J. Clin. Epidemiol..

[35] Kesteloot H., Joossens J.V. (1988). Relationship of dietary sodium, potassium, calcium, and magnesium with blood pressure. Belgian interuniversity research on nutrition and health. Hypertension.

[36] Witteman J.C., Willett W.C., Stampfer M.J., Colditz G.A., Sacks F.M., Speizer F.E., Rosner B., Hennekens C.H. (1989). A prospective study of nutritional factors and hypertension among US women. Circulation.

[37] Ascherio A., Hennekens C., Willett W.C., Sacks F., Rosner B., Manson J., Witteman J., Stampfer M.J. (1996). Prospective study of nutritional factors, blood pressure, and hypertension among US women. Hypertension.

[38] Ascherio A., Rimm E.B., Giovannucci E.L., Colditz G.A., Rosner B.A., Willett W.C., Sacks F., Stampfer M.J. (1992). A prospective study of nutritional factors and hypertension among US men. Circulation.

[39] He K., Liu K., Daviglus M.L., Morris S.J., Loria C.M., Van Horn L., Jacobs D.R., Savage P.J. (2006). Magnesium intake and incidence of metabolic syndrome among young adults. Circulation.

[40] Song Y., Sesso H.D., Manson J.E., Cook N.R., Buring J.E., Liu S. (2006). Dietary magnesium intake and risk of incident hypertension among middle-aged and older US women in a 10-year follow-up study. Am. J. Cardiol..

[41] Peacock J.M., Folsom A.R., Arnett D.K., Eckfeldt J.H., Szklo M. (1999). Relationship of serum and dietary magnesium to incident hypertension: The Atherosclerosis Risk in Communities (ARIC) Study. Ann. Epidemiol..

[42] Charlton E., Steyn K., Levitt N.S., Zulu J.V., Jonathan D., Veldman F.J., Nel J.H. (2005). Diet and blood pressure in South Africa: Intake of foods containing sodium, potassium, calcium, and magnesium in three ethnic groups. Nutrition.

[43] Townsend M.S., Fulgoni V.L., Stern J.S., Adu-Afarwuah S., McCarron D.A. (2005). Low mineral intake is associated with high systolic blood pressure in the Third and Fourth National Health and Nutrition Examination Surveys: Could we all be right?. Am. J. Hypertens..

[44] Verma H., Garg R. (2017). Effect of magnesium supplementation on type 2 diabetes associated cardiovascular risk factors: A systematic review and meta-analysis. J. Hum. Nutr. Diet..

[45] Jee S.H., Miller E.R., Guallar E., Singh V.K., Appel L.J., Klag M.J. (2002). The effect of magnesium supplementation on blood pressure: A meta-analysis of randomized clinical trials. Am. J. Hypertens..

[46] Quamme G.A. (2008). Recent developments in intestinal magnesium absorption. Curr. Opin. Gastroenterol..

[47] Saris N.E., Mervaala E., Karppanen H., Khawaja J.A., Lewenstam A. (2000). Magnesium. An update on physiological, clinical and analytical aspects. Clin. Chim. Acta.

[48] Shils M.E. (1969). Experimental production of magnesium deficiency in man*. Ann. N. Y. Acad. Sci..

[49] Quamme G.A. (1997). Renal magnesium handling: New insights in understanding old problems. Kidney Int..

[50] Barbagallo M., Dominguez L.J. (2007). Magnesium metabolism in type 2 diabetes mellitus, metabolic syndrome and insulin resistance. Arch. Biochem. Biophys..

[51] Monteiro C.A., Cannon G., Levy R.B., Moubarac J.-C., Louzada M.L., Rauber F., Khandpur N., Cediel G., Neri D., Martinez-Steele E. (2019). Ultra-processed foods: What they are and how to identify them. Public Health Nutr..

[52] Schnabel L., Kesse-Guyot E., Allès B., Touvier M., Srour B., Hercberg S., Buscail C., Julia C. (2019). Association between ultraprocessed food consumption and risk of mortality among middle-aged adults in France. JAMA Intern. Med..

[53] Machado P.P., Steele E.M., Levy R.B., Sui Z., Rangan A., Woods J., Gill T., Scrinis G., Monteiro C.A. (2019). Ultra-processed foods and recommended intake levels of nutrients linked to non-communicable diseases in Australia: Evidence from a nationally representative cross-sectional study. BMJ Open.

[54] Martinez Steele E., Baraldi L.G., Louzada M.L., Moubarac J.C., Mozafarian D., Monteiro C.A. (2016). Ultra-processed foods and added sugars in the US diet: Evidence from a nationally representative cross-sectional study. BMJ Open.

[55] World Health Organization Noncommunicable Diaseases. https://www.who.int/news-room/fact-sheets/detail/noncommunicable-diseases.

[56] Rico-Campà A., AMartínez-González M., Alvarez-Alvarez I., Mendonça R.D.D., De La Fuente-Arrillaga C., Gómez-Donoso C., Bes-Rastrollo M. (2019). Association between consumption of ultra-processed foods and all cause mortality: SUN prospective cohort study. BMJ.

[57] Kim H., AHu E., Rebholz C.M. (2019). Ultra-processed food intake and mortality in the USA: Results from the Third National Health and Nutrition Examination Survey (NHANES III, 1988–1994). Public Health Nutr..

[58] Eicher-Miller H.A., Fulgoni V.L., Keast D.R. (2012). Contributions of processed foods to dietary intake in the US from 2003–2008: A report of the food and nutrition science solutions joint task force of the academy of nutrition and dietetics, american society for nutrition, institute of food technologists, and international food information council. J. Nutr..

[59] Slimani N., Deharveng G., Southgate D.A.T., Biessy C., Chajès V., Van Bakel M.M.E., Boutron-Ruault M.C., McTaggart A., Grioni S., Verkaik-Kloosterman J. (2009). Contribution of highly industrially processed foods to the nutrient intakes and patterns of middle-aged populations in the European Prospective Investigation into Cancer and Nutrition study. Eur. J. Clin. Nutr..

[60] Serra-Majem L., Bes-Rastrollo M., Román-Viñas B., Pfrimer K., Sánchez-Villegas A., Martínez-González M.A. (2009). Dietary patterns and nutritional adequacy in a Mediterranean country. Br. J. Nutr..

[61] Altura B.M. (1981). Magnesium ions and contraction of vascular smooth muscles: Relationship to some vascular diseases. Fed. Proc..

[62] Altura B.M., Gebrewold A., Ising H., Gunther T. (1984). Magnesium deficiency and hypertension: Correlation between magnesium-deficient diets and microcirculatory changes in situ. Science.

[63] Turlapaty P., Altura B. (1980). Magnesium deficiency produces spasms of coronary arteries: Relationship to etiology of sudden death ischemic heart disease. Science.

[64] Machado A.R.D.C., Umbelino B., Correia M.L., Neves M.F. (2012). Magnesium and vascular changes in hypertension. Int. J. Hypertens..

[65] Iseri L.T., French J.H. (1984). Magnesium: Nature’s physiologic calcium blocker. Am. Heart J..

[66] Agus Z.S., Kelepouris E., Dukes I., Morad M. (1989). Cytosolic magnesium modulates calcium channel activity in mammalian ventricular cells. Am. J. Physiol. Physiol..

[67] Louvet L., Bazin D., Büchel J., Steppan S., Passlick-Deetjen J., Massy Z. (2015). Characterisation of calcium phosphate crystals on calcified human aortic vascular smooth muscle cells and potential role of magnesium. PLoS ONE.

[68] Jahnen-Dechent W., Ketteler M. (2012). Magnesium basics. Clin. Kidney J..

[69] Kolte D., Vijayaraghavan K., Khera S., Sica D.A., Frishman W.H. (2014). Role of magnesium in cardiovascular diseases. Cardiol. Rev..

[70] Houston M. (2011). The Role of Magnesium in Hypertension and Cardiovascular Disease. J. Clin. Hypertens..

[71] Belin R.J., He K. (2007). Magnesium physiology and pathogenic mechanisms that contribute to the development of the metabolic syndrome. Magnes. Res..

[72] Altura B.M., Altura B.T. (1991). Cardiovascular risk factors and magnesium: Relationships to atherosclerosis, ischemic heart disease and hypertension. Magnes. Trace Elements.

[73] Maier J.A., Bernardini D., Rayssiguier Y., Mazur A. (2004). High concentrations of magnesium modulate vascular endothelial cell behaviour in vitro. Biochim. Biophys. Acta.

[74] Satake K., Lee J.-D., Shimizu H., Uzui H., Mitsuke Y., Yue H., Ueda T. (2004). Effects of magnesium on prostacyclin synthesis and intracellular free calcium concentration in vascular cells. Magnes. Res..

[75] Soltani N., Keshavarz M., Sohanaki H., Asl S.Z., Dehpour A.R. (2005). Relaxatory effect of magnesium on mesenteric vascular beds differs from normal and streptozotocin induced diabetic rats. Eur. J. Pharmacol..

[76] Laurant P., Berthelot A. (1996). Endothelin-1-induced contraction in isolated aortae from normotensive and DOCA-salt hypertensive rats: Effect of magnesium. Br. J. Pharmacol..

[77] Ferrè S., Baldoli E., Leidi M., Maier J.A. (2010). Magnesium deficiency promotes a pro-atherogenic phenotype in cultured human endothelial cells via activation of NFkB. Biochim. Biophys. Acta (BBA) Mol. Basis Dis..

[78] Maier J.A. (2011). Endothelial cells and magnesium: Implications in atherosclerosis. Clin. Sci..

[79] Marques B.C.A.A., Klein M.R.S.T., Da Cunha M.R., Mattos S.D.S., Nogueira L.D.P., De Paula T., Corrêa F.M., Oigman W., Neves M.F. (2019). Effects of Oral Magnesium Supplementation on Vascular Function: A Systematic Review and Meta-analysis of Randomized Controlled Trials. High Blood Press. Cardiovasc. Prev..

[80] Laurant P., Dalle M., Berthelot A., Rayssiguier Y. (1999). Time-course of the change in blood pressure level in magnesium-deficient Wistar rats. Br. J. Nutr..

[81] Cantin M. (1970). Relationship of juxtaglomerular apparatus and adrenal cortex to biochemical and extracellular fluid volume changes in magnesium deficiency. Lab. Investig..

[82] Nadler J.L., Buchanan T., Natarajan R., Antonipillai I., Bergman R., Rude R. (1993). Magnesium deficiency produces insulin resistance and increased thromboxane synthesis. Hypertension.

[83] DeLalio L.J., Sved A.F., Stocker S.D. (2020). Sympathetic nervous system contributions to hypertension: Updates and therapeutic relevance. Can. J. Cardiol..

[84] James M.M.F.M. (1989). Use of magnesium sulphate in the anaesthetic management of phaeochromocytoma: A review of 17 anaesthetics. Br. J. Anaesth..

[85] James M.F.M., Cronje L. (2004). Pheochromocytoma Crisis: The Use of Magnesium Sulfate. Anesthesia Analg..

[86] James M.F., Beer R.E., Esser J.D. (1989). Intravenous magnesium sulfate inhibits catecholamine release associated with tracheal intubation. Anesth. Analg..

[87] Torshin I.I., Gromova O.A., Gusev E.I. (2009). Mechanisms of antistress and antidepressive effects of magnesium and pyridoxine. Zhurnal Nevrol. i psikhiatrii im. S.S. Korsakova.

[88] Caddell J., Kupiecki R., Proxmire D.L., Satoh P., Hutchinson B. (1986). Plasma catecholamines in acute magnesium deficiency in weanling rats. J. Nutr..

[89] Shimosawa T., Takano K., Ando K., Fujita T. (2004). Magnesium inhibits norepinephrine release by blocking N-type calcium channels at peripheral sympathetic nerve endings. Hypertension.

[90] Greenwood J., Nygard B., Brickey D. (2020). Effectiveness of intravenous magnesium sulfate to attenuate hemodynamic changes in laparoscopic surgery: A systematic review and meta-analysis. JBI Evid. Synth..

[91] Kalra S.S., Shanahan C.M. (2012). Vascular calcification and hypertension: Cause and effect. Ann. Med..

[92] Gorgels T.G.M.F., Waarsing J.H., De Wolf A., Brink J.B.T., Loves W.J.P., Bergen A.A.B. (2010). Dietary magnesium, not calcium, prevents vascular calcification in a mouse model for pseudoxanthoma elasticum. J. Mol. Med..

[93] Turgut F.H., Kanbay M., Metin M.R., Uz E., Akcay A., Covic A. (2008). Magnesium supplementation helps to improve carotid intima media thickness in patients on hemodialysis. Int. Urol. Nephrol..

[94] Louvet L., Büchel J., Steppan S., Passlick-Deetjen J., Massy Z. (2012). Magnesium prevents phosphate-induced calcification in human aortic vascular smooth muscle cells. Nephrol. Dial. Transplant..

[95] Kircelli F., Peter M.E., Ok E.S., Celenk F.G., Yilmaz M., Steppan S., Asci G., Passlick-Deetjen J. (2011). Magnesium reduces calcification in bovine vascular smooth muscle cells in a dose-dependent manner. Nephrol. Dial. Transplant..

[96] Montes de Oca A., Guerrero F., Martinez-Moreno J.M., Madueno J.A., Herencia C., Peralta A., Almaden Y., Lopez I., Aguilera-Tejero E., Gundlach K. (2014). Magnesium inhibits Wnt/beta-catenin activity and reverses the osteogenic transformation of vascular smooth muscle cells. PLoS ONE.

[97] Hruby A., O’Donnell C.J., Jacques P.F., Meigs J.B., Hoffmann U., McKeown N.M. (2014). Magnesium intake is inversely associated with coronary artery calcification: The Framingham Heart Study. JACC Cardiovasc. Imaging.

[98] de Boer I.H., Bangalore S., Benetos A., Davis A.M., Michos E.D., Muntner P., Rossing P., Zoungas S., Bakris G. (2017). Diabetes and hypertension: A position statement by the american diabetes association. Diabetes Care.

[99] Tsimihodimos V., Gonzalez-Villalpando C., Meigs J.B., Ferrannini E. (2018). Hypertension and diabetes mellitus: Coprediction and time trajectories. Hypertension.

[100] Lopez-Ridaura R., Willett W.C., Rimm E.B., Liu S., Stampfer M.J., Manson J.E., Hu F.B. (2004). Magnesium intake and risk of type 2 diabetes in men and women. Diabetes Care.

[101] Barbagallo M., Dominguez L. (2006). Ligia, J. Magnesium intake in the pathophysiology and treatment of the cardiometabolic syndrome: Where are we in 2006?. J. CardioMetabolic Syndr..

[102] Guerrero-Romero F., Rodríguez-Morán M. (2002). Low serum magnesium levels and metabolic syndrome. Acta Diabetol..

[103] Song Y., Ridker P.M., Manson J.E., Cook N.R., Buring J.E., Liu S. (2005). Magnesium Intake, C-reactive protein, and the prevalence of metabolic syndrome in middle-aged and older, U.S. Women. Diabetes Care.

[104] Corica F., Corsonello C.P.A.R.A.I.A., Ientile R., Cucinotta D., Di Benedetto A., Perticone F., Dominguez L.J., Barbagallo M. (2006). Serum Ionized Magnesium Levels in Relation to Metabolic Syndrome in Type 2 Diabetic Patients. J. Am. Coll. Nutr..

[105] Barbagallo M., Dominguez L.J. (2015). Magnesium and type 2 diabetes. World J. Diabetes..

[106] Mather H., Levin G. (1979). Magnesium status in diabetes. Lancet.

[107] Schnack C., Bauer I., Pregant P., Hopmeier P., Schernthaner G. (1992). Hypomagnesaemia in Type 2 (non-insulin-dependent) diabetes mellitus is not corrected by improvement of long-term metabolic control. Diabetologia.

[108] Resnick L.M., Barbagallo M., Gupta R.K., Laragh J.H. (1993). Ionic basis of hypertension in diabetes mellitus. Role of hyperglycemia. Am. J. Hypertens..

[109] Resnick L.M., Altura B.T., Gupta R.K., Laragh J.H., Alderman M.H. (1993). Intracellular and extracellular magnesium depletion in Type 2 (non-insulin-dependent) diabetes mellitus. Diabetologia.

[110] Barbagallo M., Di Bella G., Brucato V., D’Angelo D., Damiani P., Monteverde A., Belvedere M., Dominguez L.J. (2014). Serum ionized magnesium in diabetic older persons. Metabolism.

[111] Wälti M.K., Zimmermann M.B., Walczyk T., Spinas G.A., Hurrell R.F. (2003). Measurement of magnesium absorption and retention in type 2 diabetic patients with the use of stable isotopes. Am. J. Clin. Nutr..

[112] McNair P., Christensen M.S., Christiansen C., Madsbad S., Transbøl I. (1982). Renal hypomagnesaemia in human diabetes mellitus: Its relation to glucose homeostasis. Eur. J. Clin. Investig..

[113] Djurhuus M., Skøtt P., Hother-Nielsen O., Klitgaard N., Beck-Nielsen H. (1995). Insulin increases renal magnesium excretion: A possible cause of magnesium depletion in hyperinsulinaemic states. Diabet. Med..

[114] Banting F.G., Best C.H., Collip J.B., Campbell W.R., Fletcher A. (1922). Pancreatic extracts in the treatment of diabetes mellitus. Can. Med. Assoc. J..

[115] Atchley D.W., Loeb R.F., Richards D.W., Benedict E.M., Driscoll M.E. (1933). On diabetic acidosis: A detailed study of electrolyte balances following the withdrawal and reestablishment of insulin therapy. J. Clin. Investig..

[116] Aikaws J.K. (1960). Effect of glucose and insulin on magnesium metabolism in rabbits. A study with Mg28. Proc. Soc. Exp. Biol. Med..

[117] Mandon B., Siga E., Chabardes D., Firsov D., Roinel N., De Rouffignac C. (1993). Insulin stimulates Na+, Cl−, Ca2+, and Mg2+ transports in TAL of mouse nephron: Cross-potentiation with AVP. Am. J. Physiol. Physiol..

[118] Dai L.-J., Ritchie G., Bapty B.W., Kerstan D., Quamme G.A. (1999). Insulin stimulates Mg2+ uptake in mouse distal convoluted tubule cells. Am. J. Physiol. Content.

[119] Nair A.V., Hocher B., Verkaart S., Van Zeeland F., Pfab T., Slowinski T., Chen Y.-P., Schlingmann K.P., Schaller A., Gallati S. (2012). Loss of insulin-induced activation of TRPM6 magnesium channels results in impaired glucose tolerance during pregnancy. Proc. Natl. Acad. Sci. USA.

[120] Hruby A., Ngwa J.S., Renström F., Wojczynski M.K., Ganna A., Hallmans G., Houston D.K., Jacques P.F., Kanoni S., Lehtimäki T. (2013). Higher magnesium intake is associated with lower fasting glucose and insulin, with no evidence of interaction with select genetic loci, in a meta-analysis of 15 charge consortium studies. J. Nutr..

[121] Lee C.-T., Lien Y.-H., Lai L.-W., Chen J.-B., Lin C.-R., Chen H.-C. (2006). Increased renal calcium and magnesium transporter abundance in streptozotocin-induced diabetes mellitus. Kidney Int..

[122] Takayanagi K., Shimizu T., Tayama Y., Ikari A., Anzai N., Iwashita T., Asakura J., Hayashi K., Mitarai T., Hasegawa H. (2015). Downregulation of transient receptor potential M6 channels as a cause of hypermagnesiuric hypomagnesemia in obese type 2 diabetic rats. Am. J. Physiol. Physiol..

[123] Groenestege W.M.T., Hoenderop J.G., Heuvel L.V.D., Knoers N., Bindels R.J. (2006). The epithelial Mg2+ channel transient receptor potential melastatin 6 is regulated by dietary Mg^2+^ content and estrogens. J. Am. Soc. Nephrol..

[124] Chávez-Canales M., Arroyo J.P., Ko B., Vázquez N., Bautista R., Castañeda-Bueno M., Bobadilla N.A., Hoover R.S., Gamba G. (2013). Insulin increases the functional activity of the renal NaCl cotransporter. J. Hypertens..

[125] Komers R., Rogers S., Oyama T.T., Xu B., Yang C.-L., McCormick J., Ellison D.H. (2012). Enhanced phosphorylation of Na+–Cl− co-transporter in experimental metabolic syndrome: Role of insulin. Clin. Sci..

[126] Nishida H., Sohara E., Nomura N., Chiga M., Alessi D.R., Rai T., Sasaki S., Uchida S. (2012). Phosphatidylinositol 3-kinase/Akt signaling pathway activates the WNK-OSR1/SPAK-NCC phosphorylation cascade in hyperinsulinemic db/db mice. Hypertension.

[127] Sohara E., Rai T., Yang S.-S., Ohta A., Naito S., Chiga M., Nomura N., Lin S.-H., Vandewalle A., Ohta E. (2011). Acute insulin stimulation induces phosphorylation of the Na-Cl cotransporter in cultured distal mpkDCT cells and mouse kidney. PLoS ONE.

[128] Song Y., Manson J.E., Buring J.E., Liu S. (2003). Dietary magnesium intake in relation to plasma insulin levels and risk of type 2 diabetes in women. Diabetes Care.

[129] Barbagallo M., Dominguez L., Ligia J. (2010). Magnesium and aging. Curr. Pharm. Des..

[130] Suarez A., Pulido N., Casla A., Casanova B., Arrieta F.J., Rovira A. (1995). Impaired tyrosine-kinase activity of muscle insulin receptors from hypomagnesaemic rats. Diabetologia.

[131] Matsunobu S., Terashima Y., Senshu T., Sano H., Itoh H. (1990). Insulin secretion and glucose uptake in hypomagnesemic sheep fed a low magnesium, high potassium diet. J. Nutr. Biochem..

[132] Balon T.W., Gu J.L., Tokuyama Y., Jasman A.P., Nadler J.L. (1995). Magnesium supplementation reduces development of diabetes in a rat model of spontaneous NIDDM. Am. J. Physiol. Content.

[133] Fung T.T., Manson J.E., Solomon C.G., Liu S., Willett W.C., Hu F.B. (2003). The association between magnesium intake and fasting insulin concentration in healthy middle-aged women. J. Am. Coll. Nutr..

[134] Humphries S., Kushner H., Falkner B. (1999). Low dietary magnesium is associated with insulin resistance in a sample of young, nondiabetic Black Americans. Am. J. Hypertens..

[135] Von Ehrlich B., Barbagallo M., Classen H.G., Guerrero-Romero F., Mooren F.C., Rodriguez-Moran M., Vierling W., Vormann J., Kisters K. (2014). The significance of magnesium in insulin resistance, metabolic syndrome and diabetes—Recommendations of the association of magnesium research. V. |die bedeutung von magnesium für insulinresistenz, metabolisches sindrom un diabetes mellitus—Empfehlungen der gesellschaft für magnesium forschung e.V. Diabetol. Stoffwechs..

[136] Veronese N., Watutantrige-Fernando S., Luchini C., Solmi M., Sartore G., Sergi G., Manzato E., Barbagallo M., Maggi S., Stubbs B. (2016). Effect of magnesium supplementation on glucose metabolism in people with or at risk of diabetes: A systematic review and meta-analysis of double-blind randomized controlled trials. Eur. J. Clin. Nutr..

[137] Dustan H.P. (1990). Irvine Page lecture. Legacies of Irvine, H. Page. J. Hypertens. Suppl..

[138] Harrison D.G. (2013). The Mosaic Theory revisited: Common molecular mechanisms coordinating diverse organ and cellular events in hypertension. J. Am. Soc. Hypertens.

[139] Ross R. (1993). The pathogenesis of atherosclerosis: A perspective for the 1990s. Nat. Cell Biol..

[140] Barrows I.R., Ramezani A., Raj D.S. (2019). Inflammation, immunity, and oxidative stress in hypertension—Partners in crime?. Adv. Chronic Kidney Dis..

[141] Carbone F., Elia E., Casula M., Bonaventura A., Liberale L., Bertolotto M., Artom N., Minetti S., Dallegri F., Contini P. (2019). Baseline hs-CRP predicts hypertension remission in metabolic syndrome. Eur. J. Clin. Investig..

[142] Schüler R., Efentakis P., Wild J., Lagrange J., Garlapati V., Molitor M., Kossmann S., Oelze M., Stamm P., Li H. (2019). T cell-derived IL-17A induces vascular dysfunction via perivascular fibrosis formation and dysregulation of ·NO/cGMP signaling. Oxid. Med. Cell. Longev..

[143] Tomiyama H., Shiina K., Matsumoto-Nakano C., Ninomiya T., Komatsu S., Kimura K., Chikamori T., Yamashina A. (2017). The Contribution of Inflammation to the Development of Hypertension Mediated by Increased Arterial Stiffness. J. Am. Heart Assoc..

[144] Kramer J.H., Mak I.T., Phillips T.M., Weglicki W.B. (2003). Dietary Magnesium Intake Influences Circulating Pro-Inflammatory Neuropeptide Levels and Loss of Myocardial Tolerance to Postischemic Stress. Exp. Biol. Med..

[145] Mazur A., Maier J.A., Rock E., Gueux E., Nowacki W., Rayssiguier Y. (2007). Magnesium and the inflammatory response: Potential physiopathological implications. Arch. Biochem. Biophys..

[146] Malpuech-Brugère C., Nowacki W., Daveau M., Gueux E., Linard C., Rock E., Lebreton J.-P., Mazur A., Rayssiguier Y. (2000). Inflammatory response following acute magnesium deficiency in the rat. Biochim. Biophys. Acta (BBA) Mol. Basis Dis..

[147] Galland L. (1988). Magnesium and immune function: An overview. Magnesium.

[148] Bussière F., Tridon A., Zimowska W., Mazur A., Rayssiguier Y. (2003). Increase in complement component C3 is an early response to experimental magnesium deficiency in rats. Life Sci..

[149] Maier J.A., Malpuech-Brugere C., Zimowska W., Rayssiguier Y., Mazur A. (2004). Low magnesium promotes endothelial cell dysfunction: Implications for atherosclerosis, inflammation and thrombosis. Biochim. Biophys. Acta.

[150] Guerrero-Romero F., Bermudez-Peña C., Rodríguez-Morán M. (2011). Severe hypomagnesemia and low-grade inflammation in metabolic syndrome. Magnes. Res..

[151] Kim D.J., Xun P., Liu K., Loria C., Yokota K., Jacobs D.R., He K. (2010). Magnesium intake in relation to systemic inflammation, insulin resistance, and the incidence of diabetes. Diabetes Care.

[152] Konstari S., Sares-Jäske L., Heliövaara M., Rissanen H., Knekt P., Arokoski J., Sundvall J., Karppinen J. (2019). Dietary magnesium intake, serum high sensitivity C-reactive protein and the risk of incident knee osteoarthritis leading to hospitalization—A cohort study of 4953 Finns. PLoS ONE.

[153] Weglicki W.B., Mak I.T., Kramer J.H., Dickens B.F., Cassidy M.M., Stafford R.E., Phillips T.M. (1996). Role of free radicals and substance P in magnesium deficiency. Cardiovasc. Res..

[154] Kolisek M., Launay P., Beck A., Sponder G., Serafini N., Brenkus M., Froschauer-Neuhauser E., Martens H., Fleig A., Schweigel M. (2008). SLC41A1 is a novel mammalian Mg^2+^carrier. J. Biol. Chem..

[155] Yamanaka R., Tabata S., Shindo Y., Hotta K., Suzuki K., Soga T., Oka K. (2016). Mitochondrial Mg2+ homeostasis decides cellular energy metabolism and vulnerability to stress. Sci. Rep..

[156] Mastrototaro L., Smorodchenko A., Aschenbach J.R., Kolisek M., Sponder G. (2016). Solute carrier 41A3 encodes for a mitochondrial Mg(2+) efflux system. Sci. Rep..

[157] Liu M., Jeong E.-M., Liu H., Xie A., So E.Y., Shi G., Jeong G.E., Zhou A., Dudley J.S.C. (2019). Magnesium supplementation improves diabetic mitochondrial and cardiac diastolic function. JCI Insight.

[158] Liu M., Liu H., Xie A., Kang G.-J., Feng F., Zhou X., Zhao Y., Dudley S.C. (2020). Magnesium deficiency causes reversible diastolic and systolic cardiomyopathy. Biophys. J..

[159] Gout E., Rébeillé F., Douce R., Bligny R. (2014). Interplay of Mg2+, ADP, and ATP in the cytosol and mitochondria: Unravelling the role of Mg2+ in cell respiration. Proc. Natl. Acad. Sci. USA.

[160] Panov A., Scarpa A. (1996). Mg^2+^Control of Respiration in Isolated Rat Liver Mitochondria†. Biochemistry.

[161] Rodríguez-Zavala J., Moreno-Sánchez R., Rodriguez-Zavala J.S. (1998). Modulation of oxidative phosphorylation by Mg^2+^ in rat heart mitochondria. J. Biol. Chem..

[162] Kramer J.H., Mišík V., Weglicki W.B. (1994). Magnesium-deficiency potentiates free radical production associated with postischemic injury to rat hearts: Vitamin E affords protection. Free Radic. Biol. Med..

[163] Morais J.B., Severo J.S., Santos L.R., de Sousa Melo S.R., de Oliveira Santos R., de Oliveira A.R.S., Cruz K.J., do Nascimento Marreiro D. (2017). Role of magnesium in oxidative stress in individuals with obesity. Biol. Trace. Elem. Res..

[164] Calviello G., Ricci P., Lauro L., Palozza P., Cittadini A. (1994). Mg deficiency induces mineral content changes and oxidative stress in rats. Biochem. Mol. Biol. Int..

[165] Shah N.C., Liu J.-P., Iqbal J., Hussain M., Jiang X.-C., Li Z., Li Y., Zheng T., Li W., Sica A.C. (2011). Mg deficiency results in modulation of serum lipids, glutathione, and NO synthase isozyme activation in cardiovascular tissues: Relevance to de novo synthesis of ceramide, serum Mg2+ and atherogenesis. Int. J. Clin. Exp. Med..

[166] Kumar B.P., Shivakumar K. (1997). Depressed antioxidant defense in rat heart in experimental magnesium deficiency implications for the pathogenesis of myocardial lesions. Biol. Trace Element Res..

[167] Racay P. (2008). Effect of magnesium on calcium-induced depolarisation of mitochondrial transmembrane potential. Cell Biol. Int..

[168] Blomeyer C.A., Bazil J.N., Stowe D.F., Dash R.K., Camara A.K. (2016). Mg2+ differentially regulates two modes of mitochondrial Ca2+ uptake in isolated cardiac mitochondria: Implications for mitochondrial Ca2+ sequestration. J. Bioenerg. Biomembr..

[169] Chen Y., Wei X., Yan P., Han Y., Sun S., Wu K.-C., Fan D. (2009). Human mitochondrial Mrs2 protein promotes multidrug resistance in gastric cancer cells by regulating p27, cyclin D1 expression and cytochrome C release. Cancer Biol. Ther..

[170] Salvi M., Bozac A., Toninello A. (2004). Gliotoxin induces Mg2+ efflux from intact brain mitochondria. Neurochem. Int..

[171] Sponder G., Abdulhanan N., Fröhlich N., Mastrototaro L., Aschenbach J.R., Röntgen M., Pilchova I., Cibulka M., Racay P., Kolisek M. (2017). Overexpression of Na+/Mg2+ exchanger SLC41A1 attenuates pro-survival signaling. Oncotarget.

[172] Bednarczyk P., Dołowy K., Szewczyk A. (2005). Matrix Mg2+regulates mitochondrial ATP-dependent potassium channel from heart. FEBS Lett..

[173] Beavis A.D., Powers M.F. (1989). On the regulation of the mitochondrial inner membrane anion channel by magnesium and protons. J. Biol. Chem..

[174] Zoratti M., Szabò I. (1995). The mitochondrial permeability transition. Biochim. Biophys. Acta (BBA) Rev. Biomembr..

[175] Gorgoglione V., Laraspata D., La Piana G., Marzulli D., Lofrumento N.E. (2007). Protective effect of magnesium and potassium ions on the permeability of the external mitochondrial membrane. Arch. Biochem. Biophys..

[176] La Piana G., Gorgoglione V., Laraspata D., Marzulli D., Lofrumento N.E. (2008). Effect of magnesium ions on the activity of the cytosolic NADH/cytochrome c electron transport system. FEBS J..

[177] Seo Y.-W., Na Shin J., Ko K.H., Cha J.H., Park J.Y., Lee B.R., Yun C.-W., Kim Y.M., Seol D.-W., Kim D.-W. (2003). The Molecular Mechanism of Noxa-induced Mitochondrial Dysfunction in p53-Mediated Cell Death. J. Biol. Chem..

[178] Sharikabad M.N., Ostbye K.M., Brors O. (2001). Increased [Mg2+]o reduces Ca2+ influx and disruption of mitochondrial membrane potential during reoxygenation. Am. J. Physiol. Heart Circ. Physiol..

[179] Huang C.Y., Hsieh Y.L., Ju D.T., Lin C.C., Kuo C.H., Liou Y.-F., Ho T.-J., Tsai C.-H., Lin J.-Y. (2015). Attenuation of magnesium sulfate on CoCl_2_—Induced cell death by activating ERK1/2/MAPK and inhibiting HIF-1alpha via mitochondrial apoptotic signaling suppression in a neuronal cell line. Chin. J. Physiol..

[180] Ferrari R., Albertini A., Curello S., Ceconi C., Di Lisa F., Raddino R., Visioli O. (1986). Myocardial recovery during post-ischaemic reperfusion: Effects of nifedipine, calcium and magnesium. J. Mol. Cell. Cardiol..

[181] Boelens A.D., Pradhan R.K., Blomeyer C.A., Camara A.K.S., Dash R.K., Stowe D.F. (2013). Extra-matrix Mg2+ limits Ca2+ uptake and modulates Ca2+ uptake–independent respiration and redox state in cardiac isolated mitochondria. J. Bioenerg. Biomembr..

[182] Li Y., Wang J., Yue J., Wang Y., Yang C., Cui Q. (2018). High magnesium prevents matrix vesicle-mediated mineralization in human bone marrow-derived mesenchymal stem cells via mitochondrial pathway and autophagy. Cell Biol. Int..

[183] Franceschi C., Garagnani P., Vitale G., Capri M., Salvioli S. (2017). Inflammaging and ‘garb-aging’. Trends Endocrinol. Metab..

[184] Weglicki W.B., Bloom S., Cassidy M.M., Freedman A.M., Atrakchi A.H., Dickens B.F. (1992). Antioxidants and the cardiomyopathy of Mg-deficiency. Am. J. Cardiovasc. Pathol..

[185] AlGhatrif M., Wang M., Fedorova O.V., Bagrov A.Y., Lakatta E.G. (2017). The pressure of aging. Med. Clin. N. Am..

[186] Vasan R.S., Beiser A., Seshadri S., Larson M.G., Kannel W.B., D’Agostino R.B., Levy D. (2002). Residual lifetime risk for developing hypertension in middle-aged women and men: The framingham heart study. JAMA.

[187] Lakatta E.G. (2003). Arterial and cardiac aging: Major shareholders in cardiovascular disease enterprises: Part III: Cellular and molecular clues to heart and arterial aging. Circulation.

[188] Lakatta E.G., Levy D. (2003). Arterial and cardiac aging: Major shareholders in cardiovascular disease enterprises: Part II: The aging heart in health: Links to heart disease. Circulation.

[189] Lakatta E.G., Levy D. (2003). Arterial and cardiac aging: Major shareholders in cardiovascular disease enterprises: Part I: Aging arteries: A "set up" for vascular disease. Circulation.

[190] Safar M.E., Levy B.I., Struijker-Boudier H. (2003). Current perspectives on arterial stiffness and pulse pressure in hypertension and cardiovascular diseases. Circulation.

[191] Mitchell G.F., Lacourciere Y., Ouellet J.P., Izzo J.L., Neutel J., Kerwin L.J., Block A.J., Pfeffer M.A. (2003). Determinants of elevated pulse pressure in middle-aged and older subjects with uncomplicated systolic hypertension: The role of proximal aortic diameter and the aortic pressure-flow relationship. Circulation.

[192] Lakatta E.G. (2013). The reality of aging viewed from the arterial wall. Artery Res..

[193] Laurant P., Hayoz D., Brunner H., Berthelot A. (2000). Dietary magnesium intake can affect mechanical properties of rat carotid artery. Br. J. Nutr..

[194] Adrian M., Chanut E., Laurant P., Gaume V., Berthelot A. (2008). A long-term moderate magnesium-deficient diet aggravates cardiovascular risks associated with aging and increases mortality in rats. J. Hypertens..

[195] Hirohama D., Fujita T. (2019). Evaluation of the pathophysiological mechanisms of salt-sensitive hypertension. Hypertens. Res..

[196] Resnick L.M., Gupta R.K., DiFabio B., Barbagallo M., Mann S., Marion R., Laragh J.H. (1994). Intracellular ionic consequences of dietary salt loading in essential hypertension. Relation to blood pressure and effects of calcium channel blockade. J. Clin. Investig..

[197] Yang X.Y., Hosseini J.M., Ruddel M.E., Elin R.J. (1990). Blood magnesium parameters do not differ with age. J. Am. Coll. Nutr..

[198] Gullestad L., Midtvedt K., Dolva L.Ø., Norseth J., Kjekshus J. (1994). The magnesium loading test: Reference values in healthy subjects. Scand. J. Clin. Lab. Investig..

[199] Barbagallo M., Gupta R.K., Dominguez L.J., Resnick L.M. (2000). Cellular ionic alterations with age: Relation to hypertension and diabetes. J. Am. Geriatr. Soc..

[200] Galan P., Preziosi P., Durlach V., Valeix P., Ribas L., Bouzid D., Favier A., Hercberg S. (1997). Dietary magnesium intake in a French adult population. Magnes. Res..

[201] Dominguez L., Ligia J., Barbagallo M. (2017). The multidomain nature of malnutrition in older persons. J. Am. Med. Dir. Assoc..

[202] Morley J.E. (2010). Anorexia, weight loss, and frailty. J. Am. Med. Dir. Assoc..

[203] O’Shea E., Trawley S., Manning E., Barrett A., Browne V., Timmons S. (2016). Malnutrition in hospitalised older adults: A multicentre observational study of prevalence, associations and outcomes. J. Nutr. Health Aging.

[204] Kaiser M.J., Bauer J., Ms R.P.S.A., Uter W., Guigoz Y., Cederholm T., Thomas D.R., Anthony P.S., Charlton K.E., Maggio M. (2010). Frequency of malnutrition in Older Adults: A multinational perspective using the mini nutritional assessment. J. Am. Geriatr. Soc..

[205] Thomas A.J., Bunker V.W., Sodha N., Clayton B.E. (1989). Calcium, magnesium and phosphorus status of elderly inpatients: Dietary intake, metabolic balance studies and biochemical status. Br. J. Nutr..

[206] Löwik M.R., Van Dokkum W., Kistemaker C., Schaafsma G., Ockhuizen T. (1993). Body composition, health status and urinary magnesium excretion among elderly people (Dutch Nutrition Surveillance System). Magnes. Res..

[207] Costello R.B., Moser-Veillon P.B. (1992). A review of magnesium intake in the elderly. A cause for concern?. Magnes. Res..

[208] McIntosh W., Kubena K.S., Walker J., Smith D., Landmann W.A. (1990). The relationship between beliefs about nutrition and dietary practices of the elderly. J. Am. Diet. Assoc..

[209] Rosanoff A., Weaver C.M., Rude R.K. (2012). Suboptimal magnesium status in the United States: Are the health consequences underestimated?. Nutr. Rev..

[210] Hunt C.D., Johnson L.K. (2006). Magnesium requirements: New estimations for men and women by cross-sectional statistical analyses of metabolic magnesium balance data. Am. J. Clin. Nutr..

[211] Gámez C., Artacho R., Ruiz-López M.-D., Navarro M., Puerta A., López M. (1997). Serum concentration and dietary intake of Mg and Ca in institutionalized elderly people. Sci. Total Environ..

[212] Lipski P.S., Torrance A., Kelly P.J., James O.F.W. (1993). A study of nutritional deficits of long-stay geriatric patients. Age Ageing.

[213] Aghdassi E., McArthur M., Liu B., McGeer A., Simor A., Allard J.P., McGeer A. (2007). Dietary intake of elderly living in Toronto long-term care facilities: Comparison to the dietary reference intake. Rejuvenation Res..

[214] Iuliano-Burns S., Olden A., Woods J. (2013). Meeting the nutritional needs of elderly residents in aged-care: Are we doing enough?. J. Nutr. Health Aging.

[215] Lengyel C.O., Whiting S.J., Zello G.A. (2008). Nutrient inadequacies among elderly residents of long-term care facilities. Can. J. Diet. Pract. Res..

[216] Lammes E., Törner A., Akner G. (2009). Nutrient density and variation in nutrient intake with changing energy intake in multimorbid nursing home residents. J. Hum. Nutr. Diet..

[217] Vaquero M.P. (2002). Magnesium and trace elements in the elderly: Intake, status and recommendations. J. Nutr. Health Aging.

[218] Coudray C., Feillet-Coudray C., Rambeau M., Tressol J.C., Gueux E., Mazur A., Rayssiguier Y. (2006). The effect of aging on intestinal absorption and status of calcium, magnesium, zinc, and copper in rats: A stable isotope study. J. Trace Elements Med. Biol..

[219] Chrysant S.G., Chrysant G.S. (2019). Adverse cardiovascular and blood pressure effects of drug-induced hypomagnesemia. Expert Opin. Drug Saf..

[220] Hansen B.-A., Bruserud Ø. (2018). Hypomagnesemia in critically ill patients. J. Intensive Care.

[221] Almoussa M., Goertzen A., Brauckmann S., Fauser B., Zimmermann C.W. (2018). Posterior reversible encephalopathy syndrome due to hypomagnesemia: A case report and literature review. Case Rep. Med..

[222] Koiwai K., Takemi Y., Hayashi F., Ogata H., Matsumoto S., Ozawa K., Machado P.P., Monteiro C.A. (2019). Consumption of ultra-processed foods decreases the quality of the overall diet of middle-aged Japanese adults. Public Health Nutr..

[223] Guo W., Nazim H., Liang Z., Yang D. (2016). Magnesium deficiency in plants: An urgent problem. Crop J..

[224] Cakmak I., Yazıcı M.A., Tutus Y., Ozturk L. (2009). Glyphosate reduced seed and leaf concentrations of calcium, manganese, magnesium, and iron in non-glyphosate resistant soybean. Eur. J. Agron..

[225] Griffiths A.M., Cook D.M., Eggett D.L., Christensen M.J. (2012). A retail market study of organic and conventional potatoes (Solanum tuberosum): Mineral content and nutritional implications. Int. J. Food Sci. Nutr..

[226] Dickinson H.O., Nicolson D., Campbell F., Cook J.V., Beyer F.R., Ford G.A., Mason J. (2006). Magnesium supplementation for the management of primary hypertension in adults. Cochrane Database Syst. Rev..

[227] Rosanoff A., Plesset M.R. (2013). Oral magnesium supplements decrease high blood pressure (SBP > 155mmHg) in hypertensive subjects on anti-hypertensive medications: A targeted meta-analysis. Magnes. Res..

[228] Wu J., Xun P., Tang Q., Cai W., He K. (2017). Circulating magnesium levels and incidence of coronary heart diseases, hypertension, and type 2 diabetes mellitus: A meta-analysis of prospective cohort studies. Nutr. J..

[229] Altman D., Carroli G., Duley L., Farrell B., Moodley J., Neilson J., Smith D., Magpie Trial Collaboration Group (2002). Do women with pre-eclampsia, and their babies, benefit from magnesium sulphate? The Magpie Trial: A randomised placebo-controlled trial. Lancet.

[230] Fishel Bartal M., Sibai B.M. (2020). Eclampsia in the 21(st) century. Am. J. Obstet. Gynecol..

[231] Fang X., Wang H., Liu Z., Chen J., Tan H., Liang Y., Chen D. (2020). Posterior reversible encephalopathy syndrome in preeclampsia and eclampsia: The role of hypomagnesemia. Seizure.

[232] Pollock W., Peek M.J., Wang A., Li Z., Ellwood D., Homer C., Pulver L.J., McLintock C., Vaughan G., Knight M. (2019). Eclampsia in Australia and New Zealand: A prospective population-based study. Aust. N. Z. J. Obstet. Gynaecol..

[233] Winkler A.W., Smith P.K., Hoff H.E. (1942). Intravenous magnesium sulfate in the treatment of nephritic convulsions in adults. J. Clin. Investig..

[234] Joffres M.R., Reed D.M., Yano K. (1987). Relationship of magnesium intake and other dietary factors to blood pressure: The Honolulu heart study. Am. J. Clin. Nutr..

[235] Schwingshackl L., Chaimani A., Schwedhelm C., Toledo E., Pünsch M., Hoffmann G., Boeing H. (2018). Comparative effects of different dietary approaches on blood pressure in hypertensive and pre-hypertensive patients: A systematic review and network meta-analysis. Crit. Rev. Food Sci. Nutr..

[236] Busch V., Van Stel H.F., Schrijvers A.J.P., De Leeuw J.R.J. (2013). Clustering of health-related behaviors, health outcomes and demographics in Dutch adolescents: A cross-sectional study. BMC Public Health.

